# Atypical fusion among non-malignant cells: Unveiling the link between post-fusion-induced genome chaos and cancer initiation

**DOI:** 10.1186/s12935-026-04466-1

**Published:** 2026-10-13

**Authors:** Julian Weiler, Grigorios Raphael Topsis, Thomas Dittmar

**Affiliations:** Immunology and Tumor Biology, Center for Biomedical Education and Research (ZBAF), Witten/Herdecke University, Stockumer Str. 10, Witten, Germany

**Keywords:** Cell fusion, Genomic instability, Polyploidy, Cancer evolution

## Abstract

**Background:**

Atypical cell fusion events can disrupt the normal post-fusion process, initiating genome chaos through the merging of parental genomes accompanied by severe and complex gross structural changes. Consequently, unscheduled fusion events can accelerate tumorigenesis by fostering persistent genomic instability, serving as catalysts in the post-hybrid selection process and decisively contributing to the acquisition of novel and malignant properties. Understanding how hybrid cells survive after fusion and identifying specific transcriptional and phenotypic signatures that characterize hybrids across different fusion phases may provide valuable insights to counteract the evolution of cancer-initiating hybrids.

**Methods:**

Two distinct populations of H2B-fluorescence-labeled M13SV1 breast epithelial cells were cocultured to yield H2B double-fluorescence-tagged hybrid clones derived from spontaneous cell fusion. Hybrid clones were picked and propagated individually to establish early- and late-passage hybrid populations. Hybrid clone and passage-dependent cell fate studies were conducted via live-cell imaging and high-resolution 3D imaging to capture aberrant mitotic outcomes. Hybrids were evaluated for ploidy status, cell cycle DNA damage, cell/nuclear morphology and transcriptomic profiling using a set of distinct methods, including flow cytometry (ploidy/DNA damage analysis), diverse cell metrics and image processing software (cell/nuclear scoring) and RNA sequencing (transcriptome profiling). Subsequently, EMT marker expression, migration/invasion, and colony- and sphere-forming capacities were investigated.

**Results:**

Near-tetraploid hybrids undergo a hybrid clone-dependent ploidy reduction process upon long-term passaging, which is associated with cell shrinkage. Ploidy reduction was not coupled with substantial loss of DNA lesions but rather correlated with the degree of nuclear deformation. Hybrids were extremely chromosomally unstable, as evidenced by a high incidence of aberrant mitoses, nuclear pleomorphism, and elevated scores of micro- and multinucleation. Additionally, a subset of hybrids underwent ploidy reversal, characterized by endoreplication, cytokinesis failure or refusion, leading to the formation of exceptionally large or multinucleated cells. Transcriptome analysis revealed a differentially expressed gene profile of the hybrids associated with EMT, migratory aptitude and cancer-related traits, as validated by migration/invasion assays, Western Blots and colony/mammosphere assays.

**Conclusion:**

Collectively, the data highlight the catastrophic potential of atypical fusion processes, wherein widespread and large-scale genomic changes can occur at a rapid macroevolutionary pace and potentiate the risk for subclonal heterogeneity and malignancy.

**Supplementary Information:**

The online version contains supplementary material available at https://doi.org/10.1186/s12935-026-04466-1.

## Introduction

Cell fusion is a major and central biological process involved in several physiological mechanisms, such as embryogenesis, myogenesis, placentation and osteoclastogenesis [1]. Despite its pivotal role in developmental processes, aberrant cell fusion events can have severe consequences for organisms and can lead to the development of diseases or cancer [2, 3]. Within humans, cell fusion-dependent processes are strictly coordinated to provide healthy tissue function without pathological implications. This is typically achieved through multinucleated syncytia formation, where spatially separated, mitotically inactive nuclei drive tissue growth and protein synthesis while preserving tissue integrity [4–6]. In this context, the biological importance of keeping the nuclei in an unfused stage might be that nuclear fusion can cause widespread and complex genome reshuffling (genome chaos) linked with chromosomal rearrangements and gross structural genomic changes [3, 7]. It is well-known that fusion-derived tumor hybrid cells can inherit genomic and transcriptomic signatures from both fusion partners [8] and, in addition, can also acquire novel properties, such as an enhanced drug resistance, immune tolerance or migratory capacity [9–11]. The latter indicates that the gene pool of the hybrid cell population might be subject to genomic evolution, whereby massive and complex multi-genomic changes can drive the emergence of genetically distinct hybrid subclones. Only those subtypes survive and outcompete other players that have a fitness advantage and undergo positive selection. This post-hybrid selection process (PHSP) is also suspected of fostering intratumoral heterogeneity and eliciting the formation of subtypes harboring increased adaptability and resistance to genotoxic drugs or radiation [12].

Whole-genome duplication (WGD) is a very common phenomenon in solid tumors. Pan-cancer genome studies have shown that approximately 30–37% of all cancer cells within a tumor contain a tetraploid karyotype resulting from a WGD event [13, 14]. Different mechanisms are known to induce WGD, such as endocycling, mitotic slippage, endomitosis, cytokinesis failure or cell fusion [15]. It has long been assumed that WGD is a critical step in carcinogenesis since cells with multiple chromosome sets are predisposed to mitotic and chromosomal aberrations, a condition commonly known as chromosomal instability (CIN) [16, 17]. Cells with more than two chromosome sets are designated as polyploid and are typically characterized by their ability to enter a dormant, non-cycling state [18]. The absence of mitosis upon polyploidization prevents the formation of multipolar spindles, which is frequently present in cells that have undergone cell fusion or have not divided after completing the cell cycle [17]. Another striking incident is the assembly of kinetochore‒microtubule attachment errors, which can arise from competing microtubules emanating from opposite spindle poles, increasing the likelihood of chromosomal aberrations, e.g., chromosome nondisjunction, lagging chromosomes or chromosome bridges [19, 20]. In particular, the formation of merotelic kinetochore attachments upon multipolar division is associated with the emergence of lagging chromosomes that are trapped and damaged in the cleavage furrow and form their own micronuclei once cell division is completed [21, 22]. Beyond that, the defective nuclear lamina of the micronuclei further fuel DNA strand breaks (DSBs), large-scale structural rearrangements and can even cause chromothripsis [23, 24]. Polyploidy-related replication stress is another mechanism that can contribute to CIN. Replication stress can be driven by the overall lower amount of nucleotides or through the underscoring amount of replication factors, which provokes stalling and collapse of the replication fork during DNA replication and thus triggers the formation of DSBs [25, 26].

Polyploidy cell division is usually related to ploidy reduction triggered by multipolar mitosis [17, 27]. However, ploidy may also increase when polyploid cells undergo multipolar mitosis without subsequent cytokinesis [17, 28]. Another mechanism that can contribute to increased polyploidy is endocycle or endomitosis. While the endocycle comprises a cell cycle in which mitosis is completely skipped, the endomitotic cycle is characterized by only partial functional mitosis [18]. Both cell cycle variants are well-described processes that occur in the placenta, liver, heart, skin or bone marrow; ensure barrier function; protect against elevated stress or injury; and support biosynthetic processes [18, 29]. However, cell ploidy with extremely large genomes can also be found in various cancers [30, 31]. Those polyploid giant cancer cells (PGCCs) are abnormally sized cells that contain either one single oversized nucleus or multiple nuclei. Many reports highlight that PGCCs exhibit stemness features with tumor-initiating capacity and the capability to produce progeny via multipolar cell division or amitosis via nuclear budding [32–34]. In fact, PGCCs represent only a minor fraction of the tumor, but nonetheless, the number of PGCCs can drastically increase under stressful conditions. Several studies have revealed that the formation of PGCCs increased significantly under therapy-induced stress and, furthermore, can be responsible for the recurrence of tumors [35–37].

In this study, we show that homotypic cell fusion of non-transformed breast epithelial cells results in the formation of highly genomically unstable hybrid cells, as indicated by an increased frequency of mitotic and chromosomal aberrations. Long-term passaging of distinct hybrid clones revealed ploidy reduction from a near-tetraploid to a triploid and hyperdiploid genotype. Furthermore, single-cell fate tracking also revealed hybrid cells of enormous size containing either one single enlarged nucleus or were multinucleated. These giant hybrid cells divide in a multipolar fashion and undergo endocycle/endomitosis or aborted cytokinesis. These findings, along with the high proportion of DNA damage upon S- and M-phases across the passages, suggested high genetic diversity among the hybrid cell population, where an ongoing selection process provides the opportunity to generate subpopulations of hybrids with novel and malignant traits.

## Materials & methods

### Cell lines

M13SV1 human breast epithelial cells (a kind gift from James Trosko, Michigan State University, East Lansing, MI, USA) [38] were cultivated in RPMI 1640 media (PAN Biotech GmbH, Aidenbach, Germany) supplemented with 10% fetal calf serum (PAN Biotech GmbH, Aidenbach, Germany) and 100 U/mL penicillin/0.1 mg/mL streptomycin (PAN Biotech GmbH, Aidenbach, Germany). The following supplements were further added to the culture medium: 10 µg/mL recombinant human epidermal growth factor (rhEGF), 5 µg/mL human recombinant insulin, 0.5 µg/mL hydrocortisone, 4 µg/mL human transferrin and 10 nM β-estrogen (all supplements were purchased from Merck KGaA, Darmstadt, Germany). Histone labeling of M13SV1 cells was performed with the following vectors: H2B-GFP, provided by Geoff Wahl (Addgene plasmid # 11680; http://n2t.net/addgene:11680; RRID: Addgene_11680)) [39]; pH2B-mCherry-IRES-puro2, a gift from Daniel Gerlich (Addgene plasmid # 21045; http://n2t.net/addgene:21045; RRID: Addgene_21045) [40]; and pELF1a-H2B-GFP-IRES-Hyg. The vector pELF1a-H2B-GFP-IRES-HygR was generated via the In-Fusion^®^ HD Cloning Kit (Takara, Saint-Germain-en-Laye, France). Briefly, pLV-EF1a-IRES-Hyg, which was kindly provided by Tobias Meyer (Addgene plasmid # 85134; http://n2t.net/addgene:85134; RRID: Addgene_85134) [41], was used for the subcloning of the H2B-GFP DNA sequence between the EF1-alpha core promoter and the IRES sequence. The vector containing the IRES-Hygro cassette was linearized via PCR using the following primers: IRES-Hyg_fwd: 5’-GTGTCGTGAGGATCCGCG-3’ and IRES-Hyg_rev: 5’-CTGAAATGGAAGAAAAAAACTTTGAACCACTG-3. The sequence of H2B-GFP was amplified via PCR using the primers H2B-GFP_fwd: 5’-TTTCTTCCATTTCAGATGCCAGAGCCAGCGA-3’ and H2B-GFP_rev: 5’-GGATCCTCACGACACCTTGTACAGCTCGTCCATGCC-3’ (all primers were purchased from Thermo Fisher Scientific, Wesel, Germany). The correctness of the sequences of the plasmids we obtained was verified by Sanger sequencing (Eurofins Genomics, Ebersbach, Germany) via Eurofins standard sequencing primers. M13SV1 cells were stably transfected with one of the H2B-fluorescence-tag vectors by nucleofection (Lonza, Cologne, Germany) in accordance with the manufacturer’s instructions. Clonal selection of transfected M13SV1 cells was performed by adding the appropriate drug to the cell culture medium, which was 2 µg/mL puromycin (Thermo Fisher Scientific, Wesel, Germany) for M13SV1 clones containing the pH2B-mCherry-IRES-puro2 vector, 400 µg/mL G418 (Biochrom GmbH, Berlin, Germany) for M13SV1 cells expressing H2B-GFP and 200 µg/mL hygromycin B (Pan Biotech, Aidenbach, Germany) for the selection of M13SV1 clones containing the pELF1a-H2B-GFP-IRES-HygR vector. Resistant clones were screened for nuclear fluorescence, picked and subsequently propagated until a sufficient number of cells were reached for further experiments. All the cells were cultured in a humidified atmosphere at 37 °C with 5% CO_2_. The cell lines used in this study were routinely tested for possible mycoplasma contamination via a Mycoplasma PCR Detection Kit (BioCat GmbH, Heidelberg, Germany). Authentication of the parental cell lines and hybrid clones was confirmed by short tandem repeat (STR) analysis using the Cell Line Authentication service of Eurofins Genomics Europe Shared Services GmbH (Ebersberg, Germany). Since we obtained the M13SV1 cell line from a collaborative partner (see above), it is not listed in the Cellosaurus database. The M13SV1 and M13SV1-Cre STR profiles were therefore compared with previous STR profiles of this cell line, and no discrepancies were found.

### Generation of M13SV1 hybrid cells

Homotypic cell fusion of M13SV1 breast epithelial cells was conducted by coculturing M13SV-pH2B-mCherry-IRES-puro2 and M13SV1-pELF1a-H2B-GFP-IRES-HygR or M13SV1-H2B-GFP, respectively, at a ratio of 1:1 to yield fusion-derived clones expressing H2B-GFP and H2B-mCherry simultaneously. After cocultivation under antibiotic pressure of 2 µg/ml puromycin and 200 µg/ml hygromycin/400 µg/ml G418 for up to 21 days, we were able to pick single fusion-derived colonies originating from spontaneous fusion of M13SV1 breast epithelial cells. M13SV1 hybrid cells derived from M13SV-pH2B-mCherry-IRES-puro2 and M13SV1-pELF1a-H2B-GFP-IRES-HygR were termed M13-IRES Hybrids, whereas M13SV-pH2B-mCherry-IRES-puro2 and M13SV1-H2B-GFP cells were classified as M13-Hybrids. After successful hybrid cell selection, all hybrid clones were cultivated in RPMI media supplemented with the appropriate supplements indicated above and with the respective antibiotics puromycin (1 µg/ml), hygromycin B (100 µg/ml) or G418 (200 µg/ml).

### Flow cytometry

A total of 1 × 10^6^ cells were harvested and fixed via gentle dropwise application of ice-cold 70% ethanol and subsequently stored at -20 °C for 24 h or up to 7 days. Fixed cells were washed once with phosphate-buffered saline (PBS) (Pan Biotech, Aidenbach, Germany), and costaining for propidium iodide and γ-H2AX was performed to monitor the degree of DNA damage at distinct stages of the cell cycle. For that purpose, the cells were first permeabilized with 0.25% Triton X-100 for 15 min and then rinsed once in PBS. After this, the cells were blocked in a 5% BSA/PBS solution for 1 h at room temperature to be stained with γ-H2AX (1:400, #9718, Cell Signaling Technology Europe B.V., Frankfurt am Main, Germany) overnight in 1% BSA/PBS at 4 °C. For the IgG isotype control, a normal rabbit IgG polyclonal antibody (Merck, Darmstadt, Germany) was used. After the first antibody incubation, the cells were washed twice in PBS, and Cy-5 conjugated goat-anti-rabbit IgG (H + L) secondary antibody (1:200, Jackson ImmunoResearch, Cambridgeshire, UK) was added for an additional time of 1 h at RT in the dark. After the antibody mixture was removed, the cells were washed again twice in PBS, and the DNA was stained with FxCycle™ PI/RNase Staining Solution (Thermo Fisher Scientific, Wesel, Germany) in accordance with the manufacturer’s instructions. DNA content and γ-H2AX foci formation were evaluated via flow cytometry (FACSCalibur; Becton Dickenson, Heidelberg, Germany). The data were analyzed using the WinMDI 2.8 software (https://winmdi.software.informer.com/2.8/accessed on 1 February 2020).

### Live cell imaging

For imaging, the cells were seeded at a density of 3 × 10^5^ cells in a T25 cm^2^ flask (Sarstedt AG and Co. KG, Nümbrecht, Germany). A double thymidine block was performed to synchronize cells in the mitotic phase at a final concentration of 2 mM thymidine in complete media for 17 h. Next, the cells were washed once in PBS and the media was replaced with normal media to release the cells from G1/S-phase arrest. After 8 h, the cells were cultured again in 2 mM thymidine in a second thymidine block for an additional 16 h to ensure that almost all the cells were synchronized in the S phase. The cells were then reseeded on a 6-well plate at a density of 5 × 10^4^ cells, and after 6–8 h of release, time-lapse images of the mitotic cells were captured with an Incucyte^®^ SX5 live-cell imaging system (Sartorius AG, Göttingen, Germany). Long-term recording for up to 72 h was performed with a 20× objective. A scanning time interval of 15 min was selected to track cells for at least two mitotic rounds by using the orange and green fluorescence channels as well as the HD phase optical module. Cell fate mapping was carried out according to the protocol of Caldon et al. [42]. The mitotic outcomes of 50 distinct cells per passage were analyzed, to receive a total of 150 cells per cell line from three independent experiments.

3D live imaging was performed with a confocal laser-scanning microscope (Leica TCS SP5; Leica Microsystems, Wetzlar, Germany) to create a series of z-stack time-lapse images of cells undergoing mitosis. Approximately 2 × 10^4^ cells were seeded into 8-well µ-slides (Ibidi, Cat#: 80806, Gräfelfing, Germany). The cells were synchronized in M phase via a double thymidine block as described above. Before imaging, the media was changed to phenol red-free RPMI (PAN Biotech GmbH, Aidenbach, Germany) supplemented with 10% FCS and 20 mM HEPES pH 7.4 (PAN Biotech GmbH, Aidenbach, Germany), and the cells were stained with 100 nM Tubulin Tracker™ Deep Red (#T34077, Thermo Fisher Scientific, Wesel, Germany) for 1 h at 37 °C. Time-lapse images of z-stacks with an optical slice depth of 0.25 μm were acquired for 24 h at a constant temperature of 37 °C using a 63× high-resolution objective with immersion oil. Fluorescence image acquisition was performed via the Argon, HeNe (543 nm) and HeNe (633 nm) lasers to excite the fluorophores GFP and mCherry and the Tubulin Tracker™ Deep Red. The sequential scanning method was selected with the between-lines configuration to avoid cross-talk interference of the fluorophores. Post-processing of the confocal metadata was performed with Huygens Essential deconvolution software (Scientific Volume Imaging B.V., Hilversum, The Netherlands) using the deconvolution wizard tool. The optimized 3D time series were visualized with the Surface Render mode, Maximum Intensity Projection Renderer, or the Simulated Fluorescence Process algorithm.

### Cell proliferation analysis

Cell proliferation was analyzed via the Incucyte^®^ SX5 Live Cell Imaging system (Sartorius AG, Göttingen, Germany) by using an individually shaped mask for M13SV1 cells and for hybrid cells. For the cell proliferation study, the same plate was analyzed as indicated above for live-cell analysis. The cells were seeded in duplicate on a 6-well plate, and image acquisition was explored via a 20× objective for a total time of 72 h. Due to the heterogeneous morphology of the hybrid cells, proliferation was determined using the cell count in relation to the image area in mm. Finally, cell proliferation over time was normalized to the initial concentration at 1 h.

### Image analysis processing

For the image processing workflow, images collected from the Incucyte^®^ SX5 system were applied for the cell size analysis via the Incucyte-integrated analysis function Area Object Average available in the metric tab. To analyze the nuclear parameters, including nuclear size, eccentricity, and form factor, as well as to quantify the multinucleated cells within the cell population, the image analysis software packages Ilastik (v. 1.4.0) (https://www.ilastik.org/; European Molecular Biology Laboratory), CellProfiler (v. 4.2.4) (https://cellprofiler.org/; Broad Institute of MIT and Harvard, USA) and the ImageJ analysis tool provided in Fiji (v. 1.52 f) (https://Fiji.sc accessed on 1 February 2020) were used. First, segmentation of the fluorescence images was performed via the pixel classification option provided in Ilastik, followed by defining interphase cells against mitotic cells and cell debris via the object classification mode of the Ilastik program. Here, probability images were produced to classify whole-cell nuclei, cell debris and mitotic nuclei, which were further used for the subsequent identification of these parameters in whole image series via the batch mode tool in Ilastik. The preprocessed segmentation masks were then imported into ImageJ using the Ilastik Fiji plugin to finalize the segmentation mask by excluding mitotic cells and cell debris missed by the Ilastik operation. Finally, the final processed images were uploaded to CellProfiler for nuclear characterization. For the definition of the cell nuclei, a pixel size of 20–120 units was selected, and the minimum cross-entropy method was used to perform the object segmentation. Morphometric nuclear shape analysis was performed using the formula 4 × π × Area/Perimeter^2^ to calculate the form factor, whereby the value 1 described a complete circular object and objects below 1 had a more irregular form. The nuclear eccentricity was calculated over a range from 0 to 1, in which 0 represents a uniform circle and 1 represents a more stretched/elongated shape. To compute the frequency of multinucleated cells, the number of nuclei in the direct neighborhood was determined.

### Micronuclei analysis

The ratio of micronuclei per cell as well as the micronuclei size was determined via CellProfiler software. Here, high-brightness nuclear fluorescence images were imported into CellProfiler using a typical diameter of 2–13 pixels, corresponding to < 50 μm². A preprocessing analysis was performed that fits the parameters to create the pre-segmentation mask of detected micronuclei. The segmented images were loaded in ImageJ and manually processed to exclude nuclear debris and nonspecific dye residues. The final micronuclei segmentation images were input again into CellProfiler and analyzed using the same parameters as before. The frequency of detected micronuclei was set in relation to the total cell number to evaluate the percentage of cells with micronuclei.

### Immunocytochemical staining

The cells were seeded at a concentration of 2 × 10^4^ per well in a chamber slide (Thermo Fisher Scientific GmbH, Schwerte, Germany) at 37 °C and 5% CO_2_. Then, the cells were fixed with 4% paraformaldehyde (Affymetrix, Ohio, USA) for 15 min at RT and washed twice with PBS. After permeabilization with 0.5% Triton X-100 (AppliChem, Darmstadt, Germany) for a total time of 5 min at room temperature (RT), the cells were rinsed again three times in PBS and blocked in 1.5% BSA/PBS for 30 min at RT. The cells were incubated with the primary antibody p-H2AX139 (1:400, clone 20E3; Cell Signaling Technology Europe B.V., Frankfurt am Main, Germany; #9718) for 1 h in 1.5% BSA/PBS at RT, followed by three washes with PBS. The cells were further treated with the secondary antibody Cy-3-conjugated goat-anti-rabbit IgG (H + L) (1:500, Jackson ImmunoResearch Europe Ltd., Dianova, Hamburg, Germany) for 45 min at RT in the dark and subsequently washed three times in PBS. The chamber was removed from the slide, and the cells were mounted in Fluoromount™ aqueous mounting media (Sigma‒Aldrich, Taufkirchen, Germany). Images were captured with a TCS SP5 confocal microscope from Leica using a 63x/1.40 oil objective. Z-stack sampling was carried out with a slice depth of 0.25 μm. The reconstruction of the z-stacks was realized via the Java ImageJ plugin 3D viewer to finally create 3D projections.

### Colony formation assay

The cells were seeded at a density of 5 × 10^2^ per well in 6-well plates and cultivated in complete media for 14 days. The media were removed from the wells, and the cells were washed twice with PBS and fixed in 4% paraformaldehyde (Affymetrix, Ohio, USA) for 10 min at RT. The cells were subsequently stained with 0.5% crystal violet staining solution (Merck KGaA, Darmstadt, Germany) for 30 min at RT. Once the incubation time was complete, the cells were washed with H_2_O until only the colonies remained stained. The plates were air-dried for at least 24 h at room temperature, and images were taken. Colonies were counted manually via the image processing application Fiji (ImageJ), and the data were visualized using the GraphPad PRISM software.

### Mammosphere formation assay

M13SV1 cells and hybrid cells were harvested and seeded at a density of 1 × 10^3^ cells/well in ultralow attachment 96-well plates (Sarstedt, Nümbrecht, Germany). The cells were cultivated in mammosphere formation medium, which consisted of medium I and medium II at a ratio of 1:4. Medium I consisted of DMEM/F12 medium (Pan Biotech, Aidenbach, Germany), 6.6% B27 supplement (Thermo Fisher Scientific, Wesel, Germany) supplemented with 20 ng/mL FGF (human recombinant; Sigma‒Aldrich, Taufkirchen, Germany), 20 ng/mL EGF (human recombinant; Sigma‒Aldrich, Taufkirchen, Germany) and 0.39 µg/mL hydrocortisone (Sigma‒Aldrich, Taufkirchen, Germany). Medium II was composed of Methocult H4100 (Stem Cells Technologies, Cologne, Germany) and DMEM (Pan Biotech, Aidenbach, Germany) at a ratio of 2:3. Mammospheres were cultured for up to 10 days in an incubator at 37 °C with 5% CO_2_. Images of the Mammospheres were taken using the Incucyte^®^ SX5 Live-Cell Analysis System (Sartorius, Göttingen, Germany), and a mammosphere-specific detection mask was created via the whole-well analysis function to automatically calculate all mammospheres with a diameter not less than 60 μm.

### Scratch wound healing assay

The cells were seeded in triplicates in a 24-well plate (Sarstedt, Nümbrecht, Germany) at a density of 1.2 × 10^5^ – 2 × 10^5^ cells per well to reach a nearby confluence of 90%. The plates were incubated at 37 °C and 5% CO_2_ in complete media, and a vertical scratch was produced via a 10 µl pipette tip. The cell debris was thoroughly washed away with PBS, and the cells were cultivated in 1.5 ml of media. Wound closure was documented using the Incucyte^®^ SX5 Live-Cell Analysis System (Sartorius, Göttingen, Germany), which was set to take images every 4 h for up to 48 h. Analysis was performed with Fiji (ImageJ) software to estimate the percentage of wound closure, in which the time point at 0 h was set to 100%, and all other time intervals were set to 0 h to determine the percentage of cells that migrated into the cell-free area at the indicated time points.

### Transwell migration and invasion assays

Cell migration and invasion assays were performed using Transwell inserts (diameter 6.5 mm, 8 μm pore size; Becton Dickenson, Heidelberg, Germany) in a 24-well format. For the invasion assay, the upper chambers were pre-coated with 100 µL of a 1:4 diluted Geltrex matrix (Thermo Fisher Scientific, Wesel, Germany) pre-chilled to 4°C, and then incubated for 60 min at 37 °C to solidify the extracellular matrix barrier. For both assays, 6.5 × 10^4^ cells in 100 µL of serum-free media were carefully seeded on top of the polymerized Geltrex matrix. The lower compartment was filled with 250 µL of complete media. After incubation for 24 h at 37 °C and 5% CO_2_, the transwell inserts were removed, and the gel matrix was carefully removed with a cotton swab. The remaining cells on the lower surface of the insert were then washed in PBS and fixed with 4% paraformaldehyde (Agilent Technologies Deutschland GmbH, Waldbronn, Germany) for 15 min at room temperature. Fixed cells were washed twice with PBS and then stained with 1% crystal violet staining solution (Merck KGaA, Darmstadt, Germany) for 30 min at room temperature. Stained cells were thoroughly washed in water and air-dried overnight. Images were captured with an inverted microscope (Leica DM IRB; Leica, Wetzlar, Germany) and visualized using the Zeiss Labscope software 3.4.2 (Carl Zeiss Microscopy GmbH, Jena, Germany). In each experiment, four randomly chosen images were taken, and the average number of crystal violet-stained cells was directly analyzed using the integrated counting module of the Labscope software.

### Western blot analysis

The cells were harvested via centrifugation (10 min at 340 × g) and lysed in ice-cold RIPA buffer (50 mM Tris-HCl pH 8.0; 150 mM NaCl, 1% (v/v) NP-40, 0.5% (w/v) sodium deoxycholate, 0.1% (w/v) and sodium dodecyl sulfate) supplemented with Complete, Mini, EDTA-free Protease Inhibitor Cocktail (Sigma‒Aldrich, Taufkirchen, Germany) and Pierce Phosphatase Inhibitor Mini Tablets (Thermo Fisher Scientific GmbH, Schwerte, Germany). The samples were sonicated via a UP50H ultrasonic processor (Hielscher Ultrasonics GmbH, Teltow, Germany) for an interval of 3 × 3 using 0.5 s pulses and a recovery break on ice after each triplicate round. The protein content was determined via the Pierce™ BCA Protein Assay Kit (Thermo Fisher Scientific GmbH, Schwerte, Germany) according to the manufacturer’s instructions. Thereafter, samples were prepared for Western blotting by adding 3 × Laemmli sample buffer to the samples followed by boiling at 95 °C for 5 min. A total concentration of 40 µg protein per lane was loaded on a 10–15% polyacrylamide gel, separated at 90–120 V by sodium dodecyl sulfate‒polyacrylamide gel electrophoresis (SDS‒PAGE) and transferred to an Immobilon polyvinyl difluoride (PVDF) nitrocellulose membrane (Merck Millipore, Darmstadt, Germany) under semidry conditions. The membranes were blocked with 5% (w/v) BSA or 5% (w/v) nonfat milk powder (Applichem, Darmstadt, Germany) in Tris-buffered saline supplemented with 0.1% (v/v) Tween 20 (TBST) for 1 h at room temperature. Primary antibody incubation was performed with the following antibodies: E-Cadherin (1:1000; clone 24E10; Cell Signaling Technology Europe B.V., Frankfurt am Main, Germany; #3195), N-Cadherin (1:1000; clone 13A9; Cell Signaling Technology Europe B.V., Frankfurt am Main, Germany; #14215), Vimentin (1:1000; polyclonal; Cell Signaling Technology Europe B.V., Frankfurt am Main, Germany; #3932), p53 (1:1000; clone 1C12; Cell Signaling Technology Europe B.V., Frankfurt am Main, Germany; #2524), p21 (1:1000; clone 12D1; Cell Signaling Technology Europe B.V., Frankfurt am Main, Germany; #2947) and p73 (1:1000; clone D3G10; Cell Signaling Technology Europe B.V., Frankfurt am Main, Germany; #14620), and AJAP1 (1:1000; polyclonal; Bioss Inc., Woburn, MA, bs-13713R) overnight at 4 °C. After being washed three times in TBST, the membranes were incubated with host-specific HRP-conjugated secondary antibodies (1:3000, Cell Signaling Technology Europe B.V., Frankfurt am Main, #7074 anti-rabbit/#7076 anti-mouse) for 1 h at RT and washed again three times in TBST to remove unbound antibodies. Finally, the membranes were exposed to Pierce ECL Western blot substrate (Thermo Fisher Scientific, Wesel, Germany), and images were recorded using the Aequoria Macroscopic Imaging System (Hamamatsu Photonics Germany, Herrsching am Ammersee, Germany).

### RNA sequencing analysis

Total RNA from M13SV1-pELF1a-H2B-GFP-HygR cells and different hybrid clones was extracted via the NucleoSpin^®^ RNA Kit (Macherey Nagel, Düren, Germany) according to the manufacturer’s instructions. For the RNA-Seq study, we used three randomly selected hybrid clones that were successively passaged from passages 4–6 (designated as early-passaged hybrids) to passages 20–22 (designated as late-passaged hybrids). The detailed sample-level description is provided in Table 1. RNA integrity was determined by using an Agilent 2100 bioanalyzer (Agilent Technologies, Santa Clara, CA), and samples were sent to BGI Europe (BGI Tech Solutions, Warszawa, Poland), which fulfills the criteria (RIN value of > 8) for transcriptome sequencing. Bulk RNA sequencing was performed via a 150 bp paired-end run on the DNBSEQ platform, with an average yield of 6.78 Gb of data per sample. Quality processing to remove low-quality reads was performed, and clean reads were mapped to the human reference genome Homo_sapiens_9606.NCBI.GCF_000001405.40_GRCh38.p14.v2201 using HISAT2 (version 2.2.1), with an average alignment ratio of 98.10%. Bowtie2 (version 2.4.5) was used to align the clean reads to the reference genes, with an average alignment rate of 86.18%, resulting in an overall detection rate of 18,443 genes. Data mining was conducted on the Dr. Tom network platform of BGI (https://eu-biosys.bgi.com) to analyze significantly differentially expressed genes (DEGs) with a threshold of *q* value ≤ 0.05, log2 [Foldchange (FC)] ≥ 0.5, as well as to perform enrichment analysis based on Gene Ontology (GO), Kyoto Encyclopedia of Genes and Genomes (KEGG) and Gene Set Enrichment Analysis (GSEA). Gene expression quantification was performed using RSEM (v1.3.1) software, and gene expression clustering heatmaps across different samples were generated using pheatmap (v1.0.8). DESeq2 (v1.4.5) (or DEGseq or PoissonDis) was employed for differential gene detection, with criteria set as *q* value ≤ 0.05. To further explore possible gene functions related to phenotypic changes, we conducted GO (http://www.geneontology.org/) and KEGG (https://www.kegg.jp/) enrichment analysis on differential genes using the hypergeometric test implemented in Phyper, with a threshold of *q* value ≤ 0.05. Genes meeting this criterion were considered as significantly enriched in the candidate gene set. Principal component analysis (PCA), K-mean clustering and chromosomal gene expression visualization were annotated by using the web application software iDEP.96. (https://bioinformatics.sdstate.edu/idep96/, released July 30, 2022) Briefly, the sequencing data were downloaded from the Dr. Tom platform, transformed into the appropriate data format and then implemented into the iDEP analysis pipeline using GRCh38.p13 as the reference genome. Reads were filtered by using the default parameters of 0.5 counts per million (CPM) in at least one sample, and the edgeR algorithm was selected for the PCA and K-mean clustering analysis, with a total of 14,856 genes passing the edgeR filter (log2(CPM + 4)). The 4000 most variable genes of the dataset were selected for K-mean clustering via the elbow method to group the genes into four distinct, practical clusters. Identification of highly up- and downregulated chromosomal regions was performed with PREDA software [43], with a false discovery rate (FDR) value of 0.01. The RNA-seq data were compared with those from the TCGA and GTEx databases via the UCSC Xena web application (https://xena.ucsc.edu/*).* The GSCA online platform (http://bioinfo.life.hust.edu.cn/GSCA*)* was used to perform a pan-cancer analysis across 33 cancer types from the TCGA dataset in order to analyze the common up- and downregulated genes of the hybrid cells against diverse cancer types by calculating the normalized enrichment score. In addition, a pathway activity score of the implemented hybrid gene set was computed to examine whether it correlated with the activation or inhibition of common cancer-related pathways. The most common DEGs of the hybrid clones were determined via the UpsetR technique provided by the Dr. Tom platform, which was further used to perform enrichment analysis using the MSigDB database. Certain gene sets linked to specific pathways/hallmarks from the MSigDB analysis were picked for the construction of the protein interaction network. The STRING (version 12.0 https://string-db.org) database and Cytoscape software (v3.10.0) were used to identify hub genes, applying a confidence score > 0.7 as a significant interaction cutoff. Network construction was performed using the CytoHubba plugin via the Maximal Clique Centrality (MCC) method, by which the top 10 ranked nodes were color-highlighted from the most critical nodes in red to the least important interactions in yellow or blue. Volcano plots to visualize the top up- and downregulated genes were constructed with the R package EnhancedVolcano (v. 1.26.0), using DEGs extracted from the UpsetR results as input. The top common significantly DEGs were further used to determine their oncogenic potential using the R package tool OncoScore (https://github.com/danro9685/OncoScore). OncoScore thereby ranks the selected genes according to their association with cancer based on citation frequencies in biomedical literature [44]. The web resource TSGene 2.0 (http://bioinfo.mc.vanderbilt.edu/TSGene/) was used to screen for putative tumor suppressor genes (TSGs) on chromosome 1p36.

**Table 1:** Overview of sample groups used for comparative transcriptomic analysis

Sample ID	Clone identity	Passage number	Replicate identity	Experimental group	Abbreviation
S01	M13SV1-pH2B-IRES-GFP clone2	P5	Passage_Rep_1	Control group	Ctrl1
S02	M13SV1-pH2B-IRES-GFP clone2	P6	Passage_Rep_2	Control group	Ctrl2
S03	M13SV1-pH2B-IRES-GFP clone2	P7	Passage_Rep_3	Control group	Ctrl3
S04	M13-IRES-Hybrid1	P4	Passage_Rep_1	Early-passage	EP1
S05	M13-IRES-Hybrid1	P5	Passage_Rep_2	Early-passage	EP2
S06	M13-IRES-Hybrid1	P6	Passage_Rep_3	Early-passage	EP3
S07	M13-IRES-Hybrid1	P20	Passage_Rep_1	Late-passage	LP1
S08	M13-IRES-Hybrid1	P21	Passage_Rep_2	Late-passage	LP2
S09	M13-IRES-Hybrid1	P22	Passage_Rep_3	Late-passage	LP3
S10	M13-IRES-Hybrid2	P4	Passage_Rep_1	Early-passage	EP1
S11	M13-IRES-Hybrid2	P5	Passage_Rep_2	Early-passage	EP2
S12	M13-IRES-Hybrid2	P6	Passage_Rep_3	Early-passage	EP3
S13	M13-IRES-Hybrid2	P20	Passage_Rep_1	Late-passage	LP1
S14	M13-IRES-Hybrid2	P21	Passage_Rep_2	Late-passage	LP2
S15	M13-IRES-Hybrid2	P22	Passage_Rep_3	Late-passage	LP3
S16	M13-Hybrid2	P4	Passage_Rep_1	Early-passage	EP1
S17	M13-Hybrid2	P5	Passage_Rep_2	Early-passage	EP2
S18	M13-Hybrid2	P6	Passage_Rep_3	Early-passage	EP3
S19	M13-Hybrid2	P20	Passage_Rep_1	Late-passage	LP1
S20	M13-Hybrid2	P21	Passage_Rep_2	Late-passage	LP2
S21	M13-Hybrid2	P22	Passage_Rep_3	Late-passage	LP3

### Statistics

Statistical analysis was performed via GraphPad PRISM software 9.5.1 (https://www.graphpad.com, accessed on 1 February 2020). Multiple-group comparisons were either performed via one‐way or two-way analysis of variance (ANOVA), followed by Tukey’s post hoc test. The Kruskal‒Wallis test was used for non-normally distributed data, while Welch’s ANOVA was utilized for groups with unequal variances. A Pearson correlation analysis coupled with a two-tailed t test was performed to determine if a statistically significant linear relationship existed between two variables. A value of *P* < 0.05 was considered statistically significant.

## Results

### Hybrid cells undergo ploidy reduction without subsequent loss of DNA damage

**Fig. 1:**
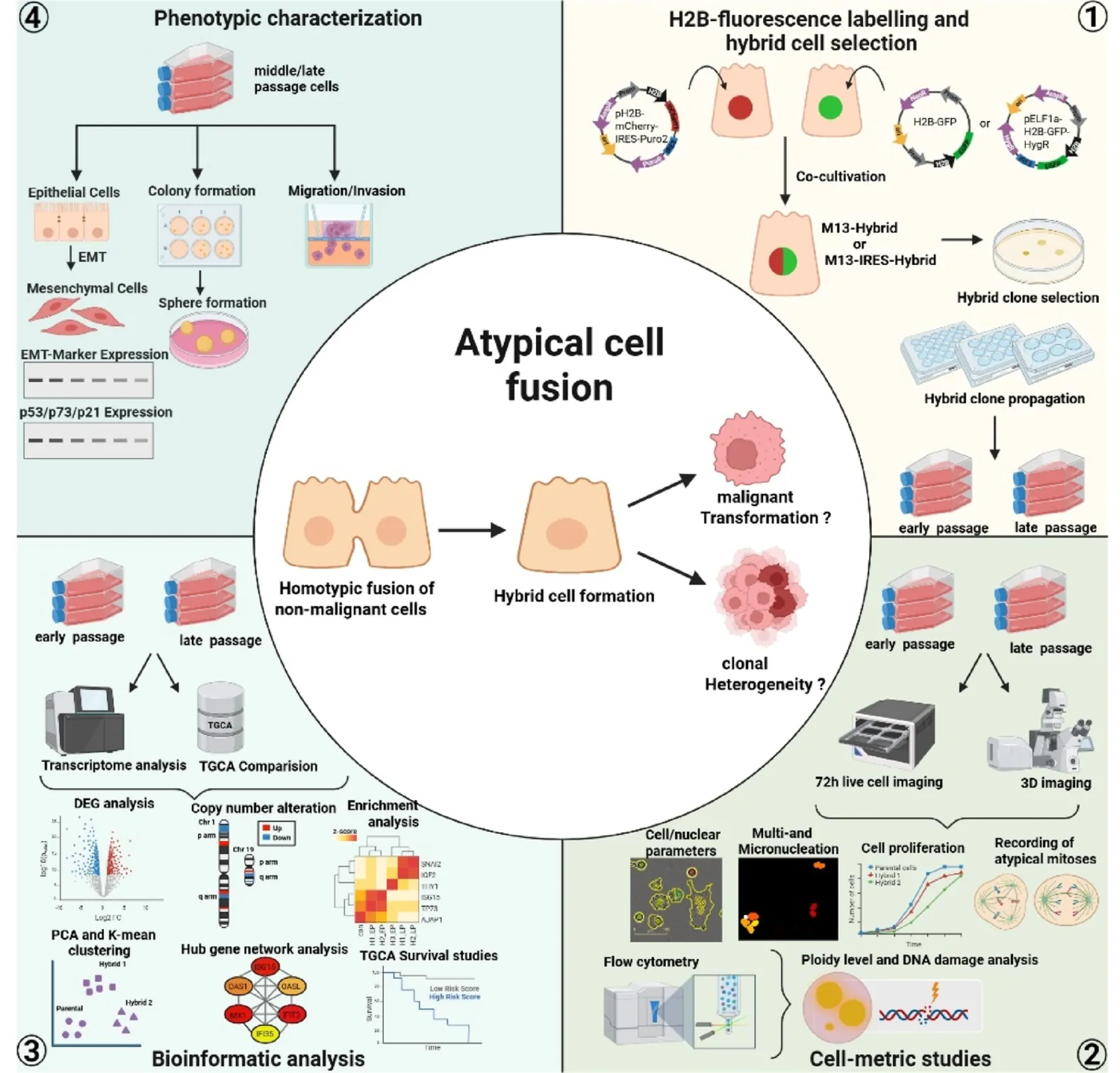
Overview of the study design concept. The experimental procedure was subdivided into four panels. The first upper right panel illustrated the experimental approach for generating double H2B-fluoresence M13SV1 hybrid cells by cocultivating of M13SV1-pH2B-mCherry-IRES-puro2 with M13SV1-pH2B-GFP or M13SV1- pELF1a-H2B-GFP-IRES-HygR, respectively. After successful hybrid clone selection and propagation, hybrids of the early passage (Passage 4–6) and late passage (Passage 20–22) were used for downstream assays. The lower right panel presents the evaluation methods to classify hybrids along their cell/nuclear parameters (area size, degree of deformation and eccentricity), determination of the micro- and multinucleation level, proliferation capacity and aberrant mitotic progression using long-term and 3D live cell imaging. In addition, ploidy level and cell-cycle DNA damage analysis was conducted via flow cytometry. The third panel displays the bioinformatic workflow to receive data of the expression profile of the hybrids and parental cells necessity to perform enrichment analysis, DEGs annotation, copy number variation and hub gene classification. Furthermore, Kaplan-Meier survival analysis from the TGCA dataset was used to compare hybrid-gene and chromosome-specific alterations with those of cancer patients. The upper left panel showed the methods used to analyze the indicated cells according to their phenotype by examining the colony and sphere formation capability, migratory and invasive aptitude or the EMT profile

**Fig. 2:**
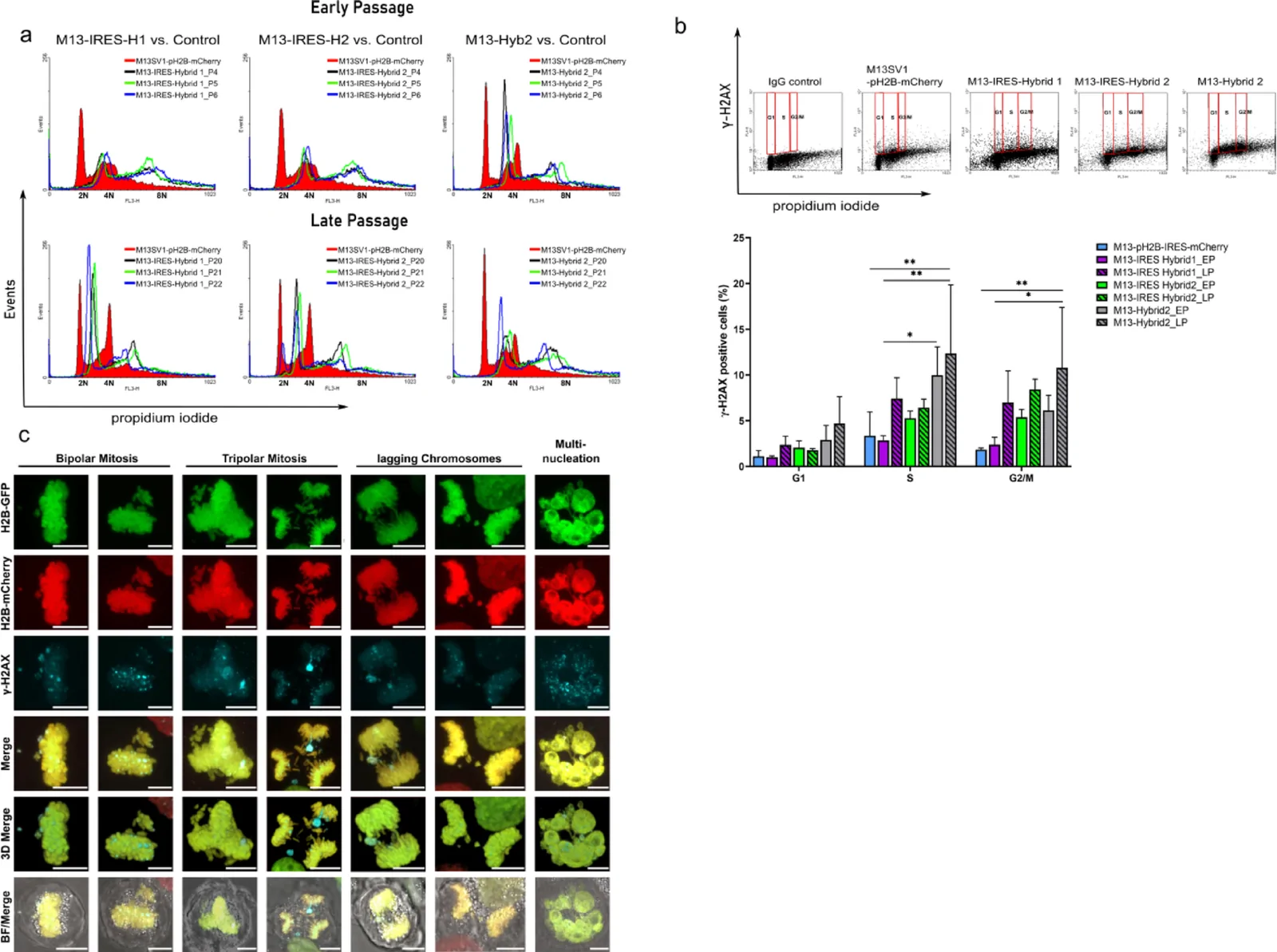
Karyotype and DNA damage analysis of fusion-derived M13SV1 hybrid cells. (**A**) The chromosomal content of three different fusion-derived M13SV1 hybrid clones was analyzed by flow cytometry using propidium iodide. At early passages, all the fusion-derived M13SV1 hybrid cells harbored a near-tetraploid karyotype, which changed across passages to a lower hybrid clone-dependent ploidy level. (**B**) Flow cytometry-based DNA damage analysis revealed that ploidy reduction did not correlate with a decrease in DNA damage. Shown are representative data of three independent measurements. For M13-IRES-Hybrid2, a mixed population developed at passage 22; therefore, the DNA damage analysis is based on two independent measurements without statistical evaluation (**C**) Z-stack images were taken via a confocal-scanning microscope (63x) with a slice depth of 0.25 μm, which were combined to create an overlay picture and a 3D projection (scale bar = 10 μm). Two-way ANOVA test and Tukey’s post hoc test were used (* *P* < 0.05, ** *P* < 0.01)

To investigate whether the hybrid cell genome underwent ploidy reduction after several rounds of cell culture, we investigated three distinct spontaneous fusion-derived hybrid clones (M13-IRES-Hybrid1, M13-IRES-Hybrid2 and M13-Hybrid2) (Fig. 1) and analyzed their DNA content at early passage (passages 4–6) and late passage (passages 20–22) by flow cytometry after propidium iodide staining (Fig. 2a). Non-fused M13SV1-pH2B-IRES-GFP cells (M13SV1) served as a control. To evaluate the degree of DNA damage that may arise in polyploid cells, the cells were costained with a fluorescence-tagged antibody targeting γ-H2AX. Compared with diploid M13SV1 control cells, almost all hybrid clones presented a near-tetraploid karyotype at low passage, which was further linked with an abundantly elevated level of DNA damage. Notably, most of the DNA damage detected was observed in the S- and M-phases of the cell cycle, in which up to 10% of the cells within the population displayed γ-H2AX foci formation as a result of damaged DNA (Fig. 2b). Approximately 15 passages later, the karyotypes of the distinct hybrid clones were re-evaluated, revealing that all hybrid clones underwent ploidy reduction in a hybrid-clone-dependent fashion. While the hybrid clones M13-IRES-Hybrid1 and M13-Hybrid2 exhibited a near-triploid karyotype, the hybrid clone M13-IRES-Hybrid2 developed at passage 22 a mixed population composed of cells that either harbored a near-triploid or near-diploid karyotype (Fig. 2a). Interestingly, ploidy reduction was not accompanied by a subsequent reduction in DNA damage in those cells. It rather seems that the extent of DNA lesions remained at an almost constant level or was even more elevated in those hybrids than in the earlier passages. Consistently, the greatest amount of DNA damage was detected during the S- and M-phases, suggesting that most DNA damage arises during the replication stage and is transmitted to the mitotic cycle without prior repair (Fig. 2b). However, DNA damage may also occur in the M phase due to chromosomal segregation defects triggered by aberrant kinetochore–microtubule interactions. To visualize DNA damage during mitosis, M13-Hybrid2 cells were investigated under a confocal laser scanning microscope (Fig. 2c). Cells were first synchronized by a double thymidine block, and mitotic cells were analyzed 8–12 h after release from G1/S-phase arrest. γ-H2AX was used as a marker for DNA damage. In addition to bipolar mitoses, we also observed chromosomal and mitotic aberrations, such as multipolar mitosis, lagging chromosomes or extrachromosomal DNA in M13-Hybrid-2 cells (Fig. 2c). Notably, γ-H2AX foci formation was seen in all dividing cells, which is indicative for a high amount of DNA damage and in good agreement with flow cytometry data (Fig. 2b). Similarly, we also found multinucleated M13-Hybrid2 cells whose nuclei were misshaped, lobulated or indicative of the formation of nuclear blebs or micronuclei (Fig. 2c).

### M13SV1 hybrid cells undergo aberrant mitoses more frequently

**Fig. 3:**
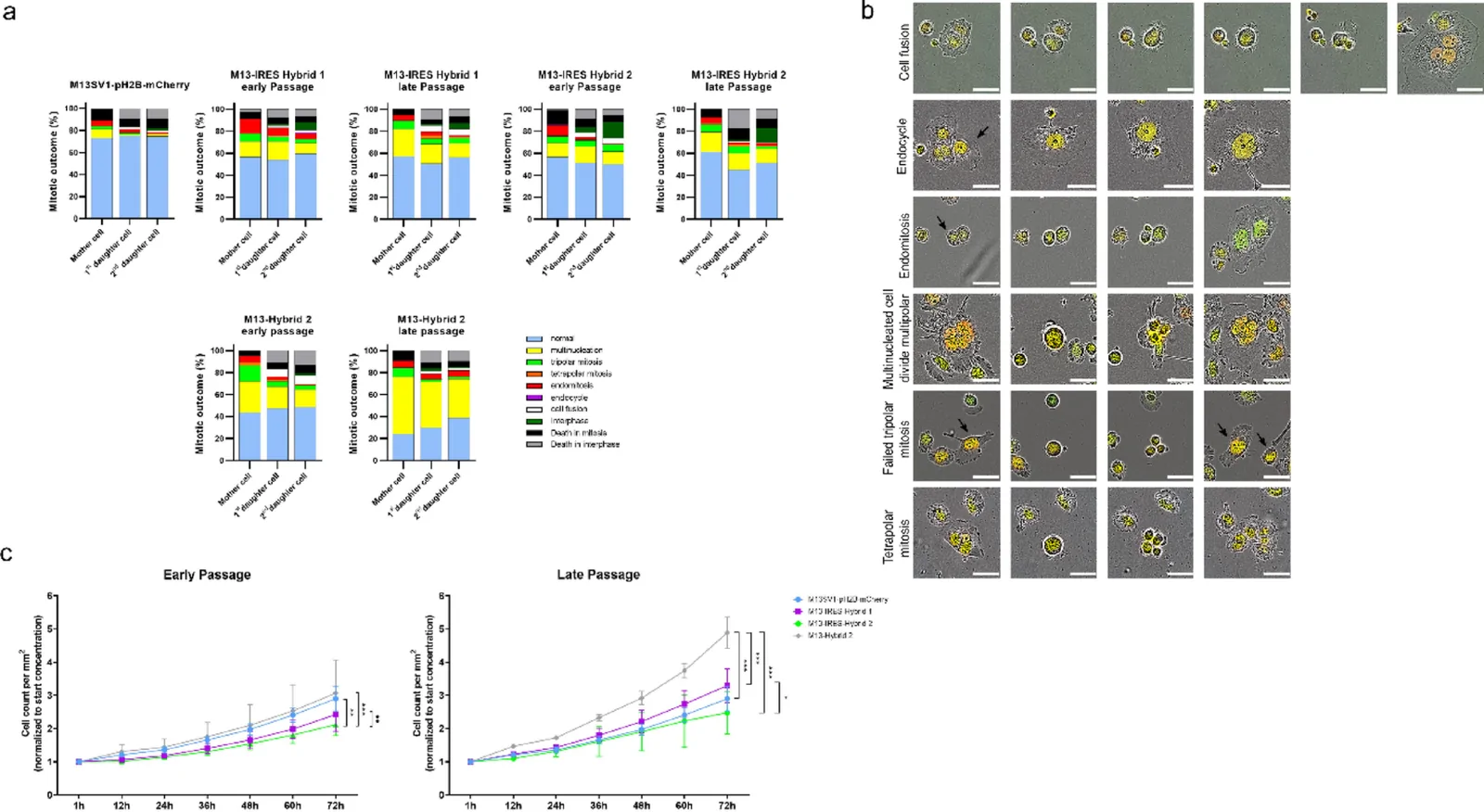
The frequency of mitotic aberrations was elevated in M13SV1 hybrid cells. (**A**) Time-lapse imaging was performed for up to 72 h to track 150 individual cell fates of the mother cell and the next generation. The cells were synchronized at the G1/S phase via a double thymidine block, released from G1/S-phase arrest, and after 6–8 h, the mitotic outcome of 50 distinct cells per passage (early passage = passages 4–6; late passage= passages 20–22) was analyzed to capture the distribution of mitotic events in a stacked column chart. In contrast to that in parental cells, the frequency of mitotic aberrations was largely increased in hybrid cells, which was associated with abnormalities such as multipolar mitoses, refusion or endomitosis. (**B**) Representative images of single cell fates acquired with the Incucyte system to illustrate mitotic aberrations in M13SV1 hybrid cells (scale bar = 50 μm). (**C**) The proliferation of early- and late-propagating hybrid clones was analyzed for 72 h and compared to that of diploid parental M13SV1-pH2B-mCherry cells. While all hybrid clones of the early passage displayed a lower proliferation capacity than the parental cells did, the proliferation capacity largely increased in the late-passage hybrid clones and surpassed the proliferation of the parental cells. Shown are representative data of three independent measurements. Two-way ANOVA test and Tukey’s post hoc test were used (* *P* < 0.05, ** *P* < 0.01, *** *P* < 0.001)

Given the large number of mitotic aberrations occurring within the hybrid clone cell populations, we next quantified the percentage of aberrant cell divisions by tracing the single-cell fates of hundreds of cells across two generations (Fig. 3a-b). Compared with diploid parental M13SV1 cells, hybrid clones presented an overall greater number of mitotic aberrations, including multipolar cell division, endoreplication, refusion or failed cytokinesis (Fig. 3a-b). In addition, high-resolution live-cell imaging further confirmed mitotic and spindle-dependent segregation abnormalities, such as lagging chromosomes and the assembly of multipolar spindles (Supplementary movies S1-S2). Aberrant mitotic outcomes were also observed in diploid M13SV1 cells, whereby one-fifth of the cells presented mitotic cell division errors (Fig. 3a). However, most of the reproduced daughter cells that originated from aberrant mitosis were nonviable and either died in interphase or during the next mitotic cell division. Conversely, hybrid clone cells showed a broader spectrum of mitotic abnormalities, ranging from 40 to 60% of all enumerated mitotic outcomes. Notably, the frequency of hybrid cell-derived progeny originated by aberrant mitoses was higher compared to diploid M13SV1 cells (Fig. 3a).

### Hybrid cells regain an elevated proliferation capacity across passages

Because the hybrid clone cells exhibited a higher frequency of DNA damage (Fig. 2b), and of chromosomal and mitotic aberrations (Fig. 3a), we wondered whether the hybrid clone cell populations still exhibit a proliferative capacity. By comparing early and late hybrid clone cells, we found that hybrid cells at later passages exhibited an increased proliferation capacity compared with early-passaged hybrid cells (Fig. 3c). The proliferation of the early-passage hybrid clone cells either remained behind the proliferation capacity of the diploid control cells or was at a comparably high level. However, this changed at late passages, whereby two out of the three hybrid clones regained their proliferation capacity, resulting in elevated cell proliferation (Fig. 3c). Compared with the other two hybrid clones, only the hybrid clone M13-IRES-Hybrid2 exhibited a lower proliferation capacity, with a similar proliferation rate to that of the diploid parental cells. Therefore, we assume that hybrid cells that have been generated recently might be more prone to prolonged mitosis than late-passaged hybrids that have undergone ploidy reduction. Additionally, it is also possible that genomic changes within the hybrid cell population may have manifested across passages, which might be permissive for the proliferation of late-passaged hybrid cells.

### Long-term cell cultivation is associated with a reduction in cell and nuclear size in the hybrid cell population

**Fig. 4:**
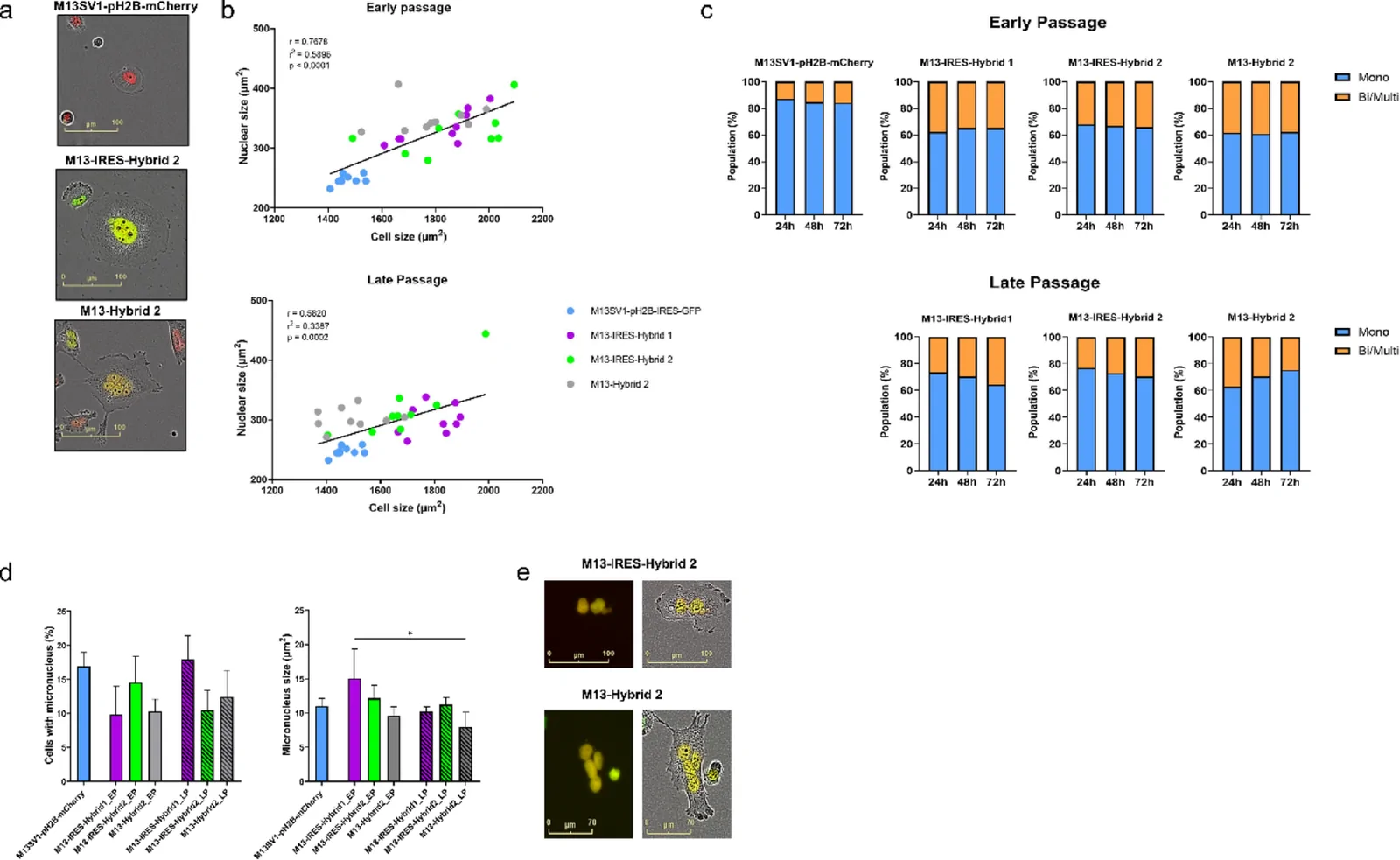
Cell metric studies revealed hybrid- and passage-dependent differences in cell/nuclear size as well as in multi- and micronucleation. (**A**) Overlay of phase-contrast and fluorescence images from the Incucyte system revealed nuclear and cell size-specific differences between the parental cells and the Hybrids. (**B**) Linear regression analysis confirmed that the cell size and nuclear size enlargement of the hybrid cells increased almost equally. A reduction in the size of the cell and nucleus was detected in late-passage hybrid cells, whereby the size of the M13-Hybrid 2 clone was near the size of the diploid control cells, but the nucleus area was still larger than that of the diploid parental cells. A Pearson correlation analysis with a two-tailed t test was used. (**C**) Bi and multinucleated cells within the hybrid cell population were identified and evaluated. While the frequency of multinucleation in early-passaged hybrids remained constant, the multinucleation rate changed in late-passage hybrids over the time of cultivation in a hybrid-clone-dependent manner. (**D**) The number of micronuclei-bearing cells and the average size of the micronuclei were analyzed using a cutoff size of < 50 µm^2^. No significant difference in the number of micronucleated cells was observed between control and hybrid cells, regardless of the passage number. However, early-passage hybrids displayed larger micronuclei than late-passage hybrids did. A one-way ANOVA test for three independent experiments coupled with a Tukey’s post hoc test were used (* *P* < 0.05) (**E**) Merged fluorescence images of hybrid cells with micronuclei display hybrid cells with several tiny micronuclei and hybrid cells with relatively large micronuclei. Shown are representative data of three independent measurements

Morphometric studies were conducted to analyze the cell size and nuclear size of early- and late-passage hybrid cells. The aim of this approach was to investigate whether hybrid cells derived by cell fusion remained consistently larger than diploid cells or whether hybrid cells underwent cell/nuclear shrinkage as an adaptation to the reduced ploidy level. An increase in cell size, which was tightly connected with nuclear enlargement, was observed in all the hybrid clones (Fig. 4a-b; Supplementary File S1). Apart from this, we observed that the cell and nuclear sizes varied among the hybrid clones and passages. Linear regression analysis (Fig. 4b) and calculation of the nuclear-to cytoplasmic ratio (Supplementary File S1) suggest a possible positive correlation between the size of the cell nuclei and an the overall cell size. Indeed, a stronger linear nuclear-to-cell size effect was observed in all early-passaged hybrid clone cells (*r* = 0.7678; r^2^ = 0.5896; *p* < 0.0001) compared to late-passaged hybrid clone cells (*r* = 0.5820; r^2^ = 0.3387; *p* = 0.0002), indicating increased cellular heterogeneity in later passages (Fig. 4b). Upon closer inspection, we noted that neither the cell size nor the nuclear size increased in a proportional fashion, as expected for cells that had undergone WGD. Compared with unfused diploid M13SV1 cells, hybrid clone cells from early passages were enlarged by only 15–40%, and the average hybrid clone cell size ranged from 1700 µm^2^ to 2100 µm^2^, whereas the mean cell size of normal diploid M13SV1 cells was about 1500 µm^2^. On the other hand, nuclear area measurements revealed that the increase in nuclear size was slightly greater than the increase in the cell size of all hybrid clones. Notably, we detected an increase of 20–60%, ranging from 300 µm^2^ in size to 400 µm^2^, compared with the average nuclear area of 250 µm^2^ in the diploid M13SV1 cells (Fig. 4b; Supplementary File S1). Comparative analysis with late-passage hybrid clone cells derived from former passages revealed that a continuous decrease in the cell and nuclear areas has occurred, which was hybrid clone dependent. M13-Hybrid2 cells showed the largest reduction in cell size, with an average size of approximately 1500 µm^2^, which exactly matched the size of the diploid control cells. However, the nuclear area of the late-passage M13-Hybrid2 cells was still enlarged and had an average size of > 300 µm^2^. For the hybrid clones M13-IRES-Hybrid-1 and − 2, cell area shrinkage was less striking at late passages, with a reduction to only 1600 µm^2^-1900 µm^2^, and was thereby only 13–25% larger than that of the diploid M13SV1 parental cells. Differences in nuclear size among the late-passage hybrid clones were also detected. Here, the nuclei of the M13-IRES-Hybrid2 cells were generally smaller than the nuclei of the other clones, with an average size of < 300 µm^2^. Interestingly, the cell and nuclear size areas increased slightly during the time of cell cultivation between 24 and 72 h, which was observed only in early-passaged hybrids (Supplementary File S1). This might be attributed to putative genome enlargement due to mechanisms such as endoreduplication or hybrid refusion (Supplementary movies S4-S9). The data obtained show that all three hybrid clones underwent cell size and nuclear shrinkage across the passages, although the extent of reduction was different among the hybrid clones. Considering the karyotype analysis (Fig. 2b), our data indicate that genome downsizing across passages was correlated with a reduction in the nuclear area. For example, the emergence of hyperdiploid and near-triploid population of M13-IRES-Hybrid2 cells at late passages was also associated with a reduction in the nuclear size of those cells. However, the near-tetraploid karyotype was not consistent with the nuclear size of the early-passaged hybrid clones, suggesting that genome doubling may not be necessarily correlated with the nuclear size of the cells.

### The frequency of multinucleated cells is more increased in the hybrid cell population

We next evaluated the level of multinucleation among the hybrid clones and between early- and late-passage hybrids. On the basis of the high frequency of aberrant mitoses indicated in the cell generation map, we determined the grade of multinucleation by using the same live-cell records. As shown in Fig. 4c, the proportion of multinucleated cells was consistently elevated in all hybrid clones, with an average multinucleation score of approximately 30–40%. This was twice as high as that observed for diploid M13SV1 cells, with approximately 15–20% multinucleated cells. We also analyzed the amount of multinucleation at 24 h intervals to investigate whether the frequency of multinucleation changed across generations. Interestingly, early-passaged cells constituted a consistently higher proportion of multinucleated cells in a hybrid clone-dependent manner. Here the M13-IRES-Hybrid1 and M13-Hybrid2 hybrids displayed a frequency of almost 40% and the M13-IRES-Hybrid2 cells constituted approximately 30% of multinucleated cells (Fig. 4c). Unlike those of early-passage hybrids, the number of multinucleated cells varied more sharply every 24 h in late-passage hybrids. For M13-IRES-Hybrid1, the incidence of multinucleated cells increased every 24 h interval, with a 30% multinucleation score after 24 h (Fig. 4c). Thereby, the highest number of multinucleated cells was detected at 72 h of incubation, with approximately 40% multinucleated cells (Fig. 4c). A more gradual increase in multinucleated cells per 24 h was determined for the M13-IRES-Hybrid2 cells, from a 25% multinucleated fraction to a 30% multinucleated fraction after 72 h of incubation (Fig. 4c). Only the hybrid clone M13-Hybrid2 showed a decrease in multinucleation over time, which initially reached 40% after 24 h, then decreased to 30% after 48 h and finally reached a total multinucleation rate of 25% after 72 h of incubation (Fig. 4c). The data obtained confirmed our results of the single-cell fate recording study (Fig. 3a), whereby failure of mitosis, refusion or endomitosis steadily contributed to the generation of multinucleated hybrid cells within the hybrid cell population. The dynamic changes in multinucleation at late passages might be explained by the fact that hybrids from later passages regained proliferation capacity, resulting in more dividing hybrid cells than those in early passages and thus increasing the frequency of mitotic aberrations. Although the frequency of multinucleation was similar to that detected in the single-cell fate map (Fig. 3a), the frequency of multinucleation was somewhat at odds with the results of the cell fate study. The differences might be explained by the fact that during single-cell fate mapping, only a certain number of selected cells were analyzed, just as the daughter cells originated from them, whereas in this study, the total percentage of multinucleation was determined immediately via segmentation images from all triplicates.

### Micronuclei formation in hybrids remained at a constant level across passages

The extent of mitotic aberrations within the hybrid cell populations suggested that spindle attachment errors may also provoke the formation of micronuclei. To assess the involvement of micronuclei formation as a marker for CIN in the hybrid cell population, we used a micronuclei-specific segmentation mask to detect micronuclei that were less than 1/5 the size of the primary nucleus in relation to the diploid M13SV1 cells. Micronuclei formation scoring was also used to determine whether the size of the micronuclei varied among the hybrids or across passages. Surprisingly, quantification of the average number of micronuclei per cell revealed that all hybrid clones formed micronuclei in a similar ratio as the parental M13SV1 cells did, whereby 10–15% of the cells contained at least one micronucleus (Fig. 4d). The average frequency of micronuclei in late-passage hybrid clone cells was not significantly different from that in hybrid clone cells at the early passage. For the M13-IRES-hybrid1 cells, micronuclei formation was slightly higher in the late-passage hybrids, with an average number of 17% as compared to 10% micronuclei in earlier passages (Fig. 4d). In contrast, compared with early-passage cells, late-passage M13-IRES-Hybrid2 cells harbored fewer micronuclei (on average 14%) compared to early-passage cells (on average 10%; Fig. 4d). In addition, the micronuclei formation capacity of M13-Hybrid2 cells hardly differed between early and late passages. The average frequency of cells with micronuclei was about 10–12%. Second, the average micronuclei size was analyzed, which revealed hybrid clone- and passage-dependent differences. Notably, the average size of the micronuclei was larger in early-passaged cells than in late-passaged cells, even though the size of the micronuclei detected in the distinct hybrid clones was different among themselves (Fig. 4d-e). The largest micronuclei were detected in M13-IRES-Hybrid1 cells at early passages, with an average area of 15 µm^2^ that decreased to 10 µm^2^ at higher passages. The other hybrid clones at the early passages generated micronuclei of lesser magnitude with an average size of 10–12 μm². At late passages, all hybrid clones exhibited an average micronucleus size of 10 μm², which corresponded to the micronucleus size of the diploid M13SV1 parental cells. Interestingly, the frequency of micronucleation in hybrid clones was less elevated than in diploid M13SV1 cells, despite a higher degree of DNA damage and mitotic aberrations in the hybrids. We cannot rule out that the frequency of micronuclei formation within the hybrid cell populations might be larger, for example, when the micronuclei counting mask has been fit to 1/5 of the size of the main nucleus of the hybrid cells. Since the average size of the nucleus changed immensely among the hybrid clones and passages, we opted to use a uniform micronuclei mask by setting the segmentation mask in relation to the normal nucleus size of the diploid cells. However, ploidy reduction in later passages (Fig. 2a) was closely related to the formation of micronuclei of smaller size (Fig. 4d), whereas the micronucleation-forming ability was not affected and resulted in a similar or greater capacity for micronuclei formation, which is consistent with the results of the DNA damage study.

### Nuclear deformation was more intense in the hybrids and correlated with the degree of DNA damage

**Fig. 5:**
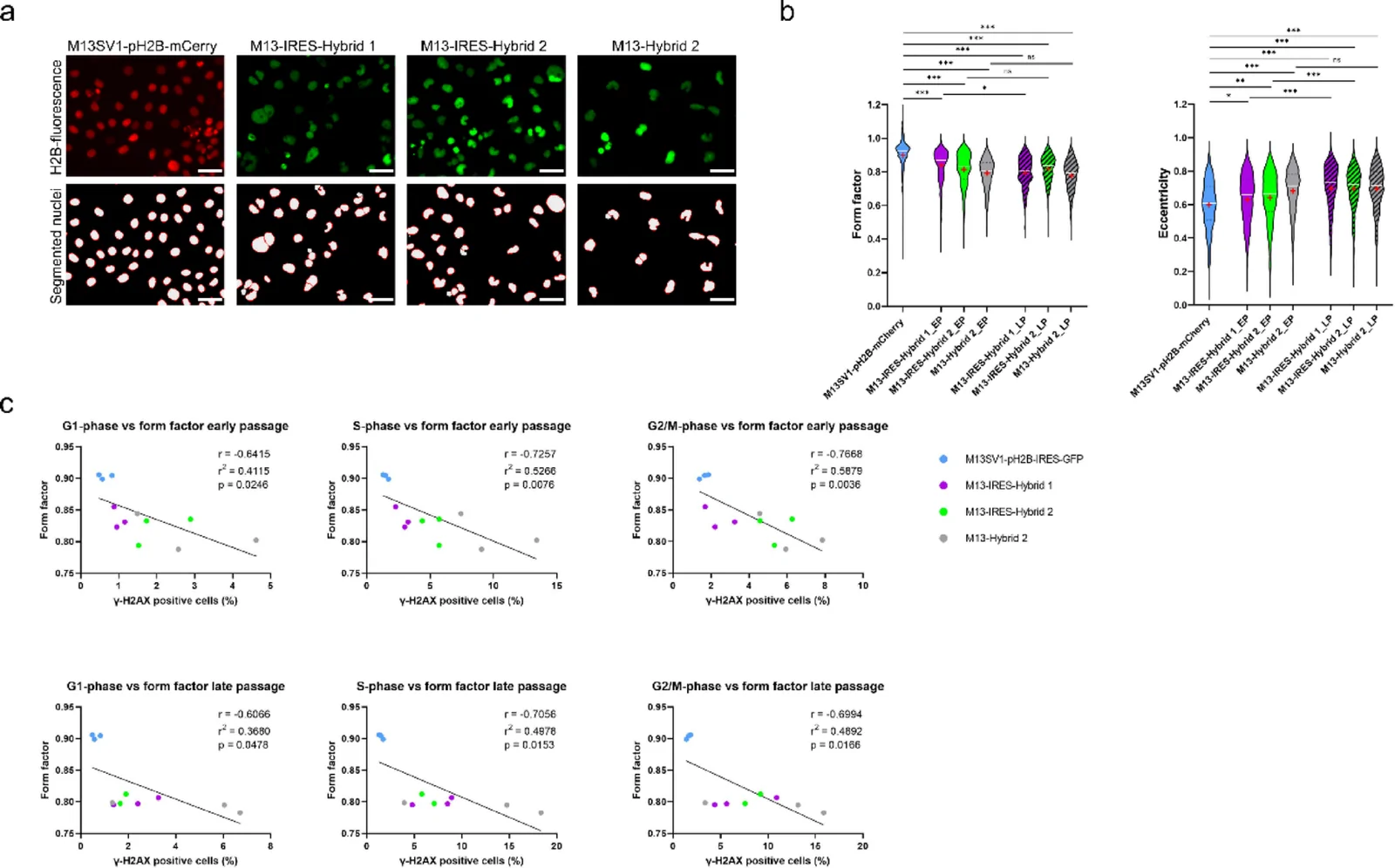
M13SV1 hybrid cells displayed a high degree of deformed nuclei. (**A**-**B**) Nuclear dysmorphia of 600 nuclei/cell lines (200 nuclei/passage; *n* = 3) were investigated by calculating the circularity of the nuclear shape, indicated as a form factor. For that reason, fluorescent chromatin images were exported via the CellProfiler pipeline, and nuclear roundness was determined using the formula 4 × π × Area/Perimeter^2^, where the value “1” represents a perfect circle and “0” indicates a fully nonround object. Hybrids at early and late passages presented markedly increased numbers of deformed nuclei, whereas the nuclei of the parental cells were rounder. In addition, the eccentricity of the nucleus was determined, as the hybrid cells harbored more enlarged nuclei than did the parental cells, which contained more oval-shaped nuclei. A one-way nested ANOVA for three independent experiments, coupled with Tukey’s post hoc test was used, and data were pooled for visualization via a violin plot. The red plus symbol indicates the mean, the white line indicates the median, and the dashed lines indicate the lower and upper quartiles (* *P* < 0.05, ** *P* < 0.01, *** *P* < 0.001, ns = not significant). (**C**) Correlation analysis revealed that deformation of the nucleus was correlated with the degree of DNA damage. Pearson correlation analysis with a two-tailed t test was used

As another parameter for CIN, we assessed the degree of nuclear deformation in early- and late-passage hybrids. For that reason, we calculated the circularity of the nuclei using the form factor and eccentricity to compare the morphology of the nuclei of the M13SV1 hybrid clone cells to that of the unfused diploid M13SV1 cells (Fig. 5a-c). All hybrid clones displayed a severe degree of nuclear deformation linked with several shape-related irregularities, such as multi-lobulation, invagination and nuclear blebbing. The extent of nuclear dysmorphia in the hybrid clones was consistent across passages, and no improvement towards a round or oval-shaped morphology at later passages was observed among the distinct hybrid clones. On the other hand, the nuclei of the diploid parental cells were typically rounder in shape with a regular and smoother surface than those of the hybrid cells (Fig. 5a-b). By examining the eccentricity factor, we found that hybrid clones of the later passages formed more elongated nuclei than early-passaged cells did. This may indicate that the nuclear architecture of the hybrid clones has changed across the passages towards a smaller and more elongated structure. The high degree of misshapen nuclei across passages indicates that CIN was constantly elevated in all hybrid clones. This result agrees with previous findings (Fig. 3a, b) and might suggest that the hybrid genome undergoes a dynamic transformation process with unexpected outcomes. To investigate the possible relationship between DNA damage formation and the number of deformed nuclei, we performed a Pearson correlation analysis. Indeed, a positive linear relationship between the number of γ-H2AX foci and nuclear deformation in hybrid cells across distinct cell cycle stages was determined (Fig. 5c). Furthermore, this correlation was significantly stronger during the S- and M-phases (*r* > 0.7; r^2^ > 0.5; *p* < 0.01) than in the G1-phase (*r* > 0.6; r^2^ < 0.5; *p* < 0.05).

### Comparative transcriptome analysis revealed hybrid and passage-dependent discrepancies

**Fig. 6:**
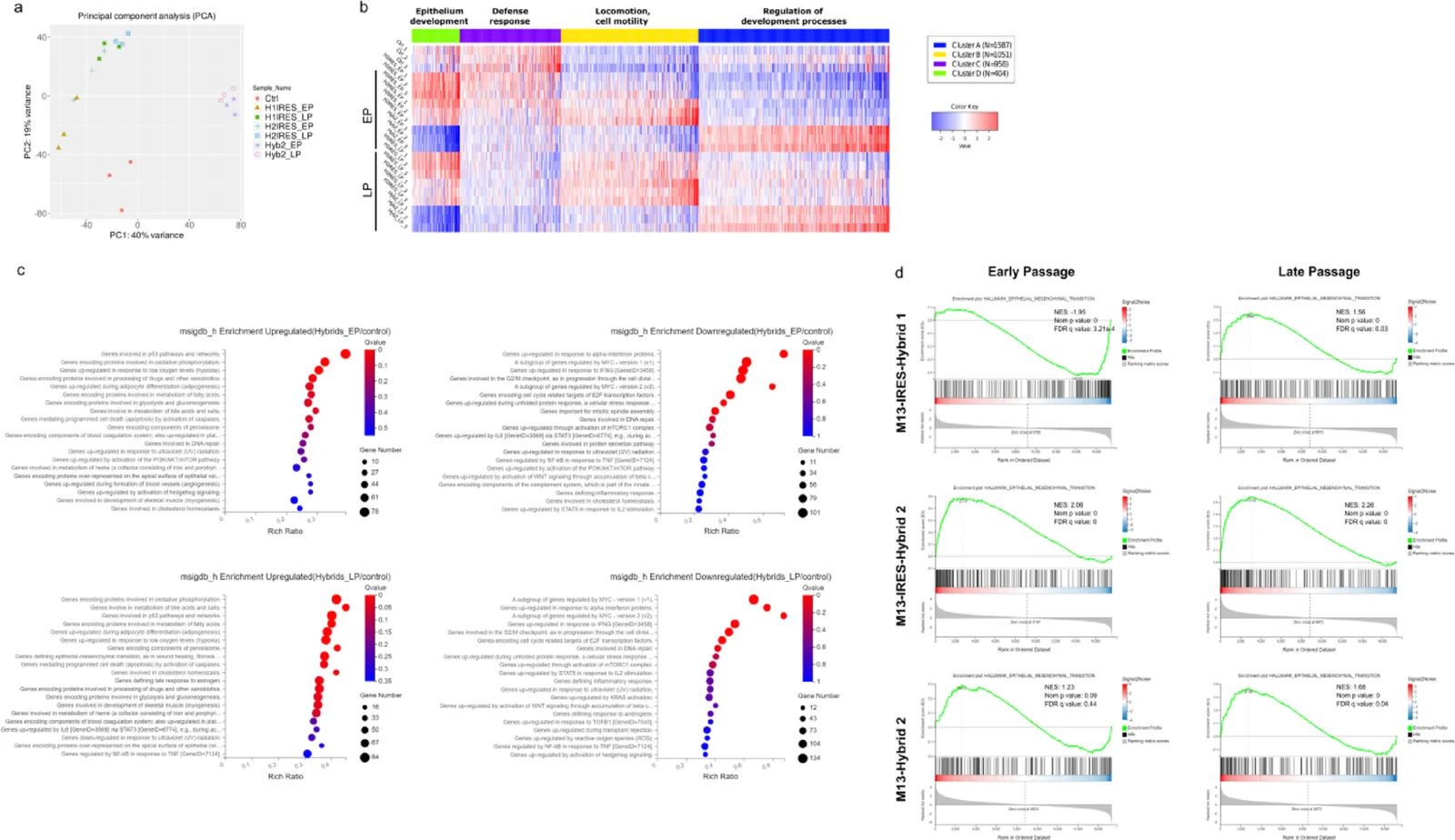
Transcriptomic profiling revealed hybrid and passage-dependent discrepancies. (**A**) Principal component analysis (PCA) of the distribution along the PC1 and PC2 axes revealed transcriptional differences between control cells and hybrid clones, as well as among the hybrid clones themselves. (**B**) K-mean clustering of the 4000 top genes in M13SV1 parental cells and hybrid clones was performed, and the genes were subdivided into four gene clusters. Each cluster represents differences in biological processes according to the GO database. (**C**) Significantly upregulated genes (log2 > 0) and downregulated genes (log2 < 0) in early- and late-passage hybrids compared with M13SV1 control cells. Bubble enrichment charts were generated via the MiSigDB database. Interestingly, genes related to p53 signaling, hypoxia and drug metabolism were upregulated in early- and late-passage hybrids, whereas EMT genes were significantly upregulated only in late-passage cells. (**D**) GSEA of EMT-related genes revealed that only early-passage M13-IRES-Hybrid1 cells presented a negative EMT enrichment score. Comparison of the NES values showed discrepancies in EMT progression among the hybrids and between early- and late-passaged cells. All data represent three independent measurements

Owing to the clearly detectable genomic instability in the hybrid cells, we were keen to determine how the gene expression profile in the distinct hybrids might change across passages. The principal component analysis (PCA) revealed high transcriptomic heterogeneity between the parental cell line and the hybrid clones M13-IRES-Hybrid2 and M13-Hybrid2, while M13-IRES-Hybrid1 cells at early passages showed more similarities with the gene expression profile of the parental M13SV1 cell line (Fig. 6a). However, transcriptome comparison via PCA revealed that the M13-IRES-Hybrid1 clone developed a more discriminative expression profile at later passages, differing markedly from that of the parental cells. We further performed a cluster analysis to allocate genes into four different clusters, which were subsequently used to classify the biological function of these gene sets by referring to the Gene Ontology database and the molecular signature database (Fig. 6b; Supplementary File S2). In accordance to PCA data, K-mean cluster analysis revealed a significantly altered gene expression profile (Fig. 6b). Moreover, the issue of a different passage-dependent gene expression pattern was much more apparent when the K-mean clustering method was used. For example, downstream enrichment analysis of the distinct clusters revealed that in the parental M13SV1 cell line more genes related to epithelium development and the defense response were significantly upregulated, whereas these genes were significantly downregulated in all hybrid clones (Fig. 6b). Similarly, all hybrid clones exhibited a transcriptomic profile distinct from that of M13SV1 parental cells. For example, both M13-IRES-Hybrid clones showed an upregulated expression profile of genes linked to locomotion, cell motility and EMT progression (Fig. 6b). Additionally, in M13-IRES-Hybrid1 cells, a passage-dependent increase in the expression of these target genes was observed, resulting in a higher expression of these genes in later passages. In contrast, M13-Hybrid2 cells exhibited a more unique gene expression profile that differed markedly from that of M13-IRES-Hybrids. Genes, which are associated with cell developmental processes and cell adhesion were found to be upregulated in M13-Hybrid2 cells (Fig. 6b). In conclusion, our data show that the merger of genetically identical cell types can give rise to the formation of genomic-instable hybrid cells, which exhibit an individual, hybrid clone-dependent transcriptomic profile that can change over the course of subclonal evolution in a dynamic manner.

### Multi-classification enrichment analyses to decipher passage-dependent differences in the hybrids

Functional enrichment analyses were carried out to identify DEGs regulating biological processes (GO_BP) and signaling pathways (KEGGs) or that were representative of molecular signatures in the hybrids (MiSigDB) (Supplementary File S3a-f). Gene ontology analysis of biological processes revealed that both early- and late-passage hybrid clone cells had an enrichment of approximately 150 genes associated with positive regulation of cell migration (Supplementary Fig. S3a-b). In contrast, only early-passage hybrid clone cells displayed a class of enriched genes that were associated with the negative regulation of cell migration. In addition, we identified more than 200 enriched genes that were associated with the negative regulation of cell proliferation and a comparative number of enriched genes related to the induction of apoptosis in both groups. KEGG pathway enrichment analysis indicated that the most significantly enriched genes, with approximately 250 genes in both compared groups, were linked with pathways in cancers (Supplementary Fig. S3c-d). Although many pathways were enriched in both groups, e.g., focal adhesion, p53 signaling, and mucin type-O-glycan biosynthesis, we also observed differences in pathways, such as RAP1 signaling, Wnt- and PI3K-Akt signaling in early-passage hybrids versus Notch signaling and glutathione metabolism in late-passage hybrid clones. Similarly, MiSigDB was used to identify gene sets indicative of molecular hallmarks in early- and late-passage hybrid clones (Supplementary Fig. S3e + f). Epithelial‒mesenchymal transition was one of the most significant and highly enriched gene sets in early- and late-passage hybrid clones, involving more than 140 genes in both groups. Among the top 20 significant clusters from MiSigDB, only two categories differed between early- and late-passage hybrid clones. In early-passage hybrid clones, a small number of genes related to Hedgehog and Notch signaling activation were determined, whereas genes correlated with cholesterol homeostasis and genes responsive to androgens were annotated in late-passage hybrid clones.

Using MiSigDB, a detailed enrichment analysis of significantly up- and downregulated genes was performed to first identify genes whose expression was upregulated or downregulated in the respective passage group (Fig. 6c), followed by gene set enrichment analysis (GSEA) to compare each hybrid clone with each other (Fig. 6d; Supplementary Table. S1). Genes that were commonly upregulated in early- and late-passage hybrid clones were related to p53 signaling, hypoxia, xenobiotic drug metabolism, glycolysis, glucogenesis and caspase-induced apoptosis. Interestingly, genes necessary for DNA repair were only upregulated in all early-passage hybrid clones, whereas genes associated with EMT were enriched only in late-passage hybrid clones (Fig. 6c). Conversely, GSEA revealed that all hybrid clones of the early- and late-passages exhibited a significantly upregulated set of genes related to EMT, except for the early-passage M13-IRES-Hybrid1 cells, which displayed a negative EMT score (Fig. 6d, Supplementary Table. S1). At later passages, the EMT-related gene signature of M13-IRES-Hybrid1 changed, resulting in the upregulation of genes indicative of EMT. By analyzing the most significantly downregulated genes, we found that the expression of cell cycle checkpoint genes involved in the G2/M transition, as well as E2F transcription factors, which are known to be relevant for the G1/S phase transition, was downregulated in early- and late-passage hybrids (Fig. 6c). However, genes related to mitotic-spindle assembly were downregulated only in all early-passage hybrid clones (Fig. 6c). Furthermore, more than 100 genes related to interferon-alpha (*IFN-α*) and interferon-gamma (*IFN-γ*) were considerably downregulated in both groups. Additional downregulated gene clusters involved in DNA repair, the inflammatory response, the *MYC1* and *MYC2* versions or the upregulation of genes required for the unfolded protein response were identified (Fig. 6c). Based on the NES, we also observed that hybrid clones presented different enriched metric scores for the same gene sets, suggesting that certain processes, pathways or phenotypes are differentially prevalent in these cells (Supplementary Table. S1). In summary, these data indicate that the transcriptomic profile of hybrid clone cells and parental M13SV1 cells changed across passages, but that certain processes, pathways and molecular hallmarks remained consistently active across passages.

**Fig. 7:**
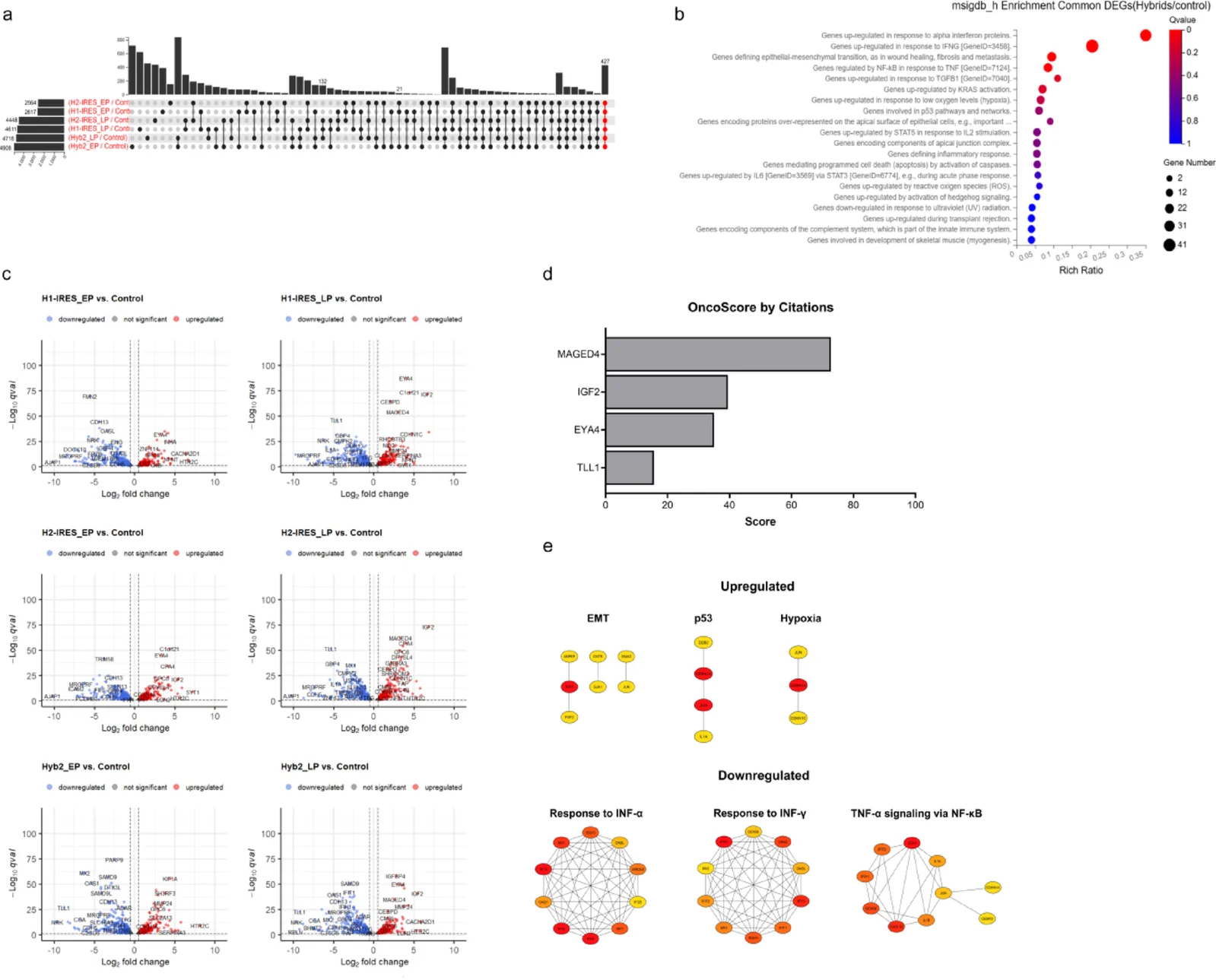
Analysis of the most common significant DEGs in hybrids. (**A**) Common DEGs of all hybrids in relation to the M13SV1 control cells were determined using the UpsetR technique (*q* value ≤ 0.05, log2FC ratio ≥ 0.5). (**B**) A total of 427 DEGs were identified and subsequently subjected to enrichment analysis via MSigDB (*q* value ≤ 0.05, log2FC ratio ≥ 0.5). (**C**) Volcano plots of common DEGs whose expression was significantly upregulated or downregulated in the hybrids vs. parental M13SV1 cells. (**D**) Literature-based ranking of genes by their putative association with cancer using the OncoScore prediction tool. (**E**) Hub gene analysis of selected pathways from the common MSigDB bubble chart. The degree of interaction is highlighted from blue to red, whereby blue indicates a low degree of gene connection and red indicates a high degree of interaction using the maximal clique centrality (MCC) operation module in CytoHubba. All data represent three independent measurements

### Common DEGs of the hybrids correlate with the expression scores of various cancer types

**Fig. 8:**
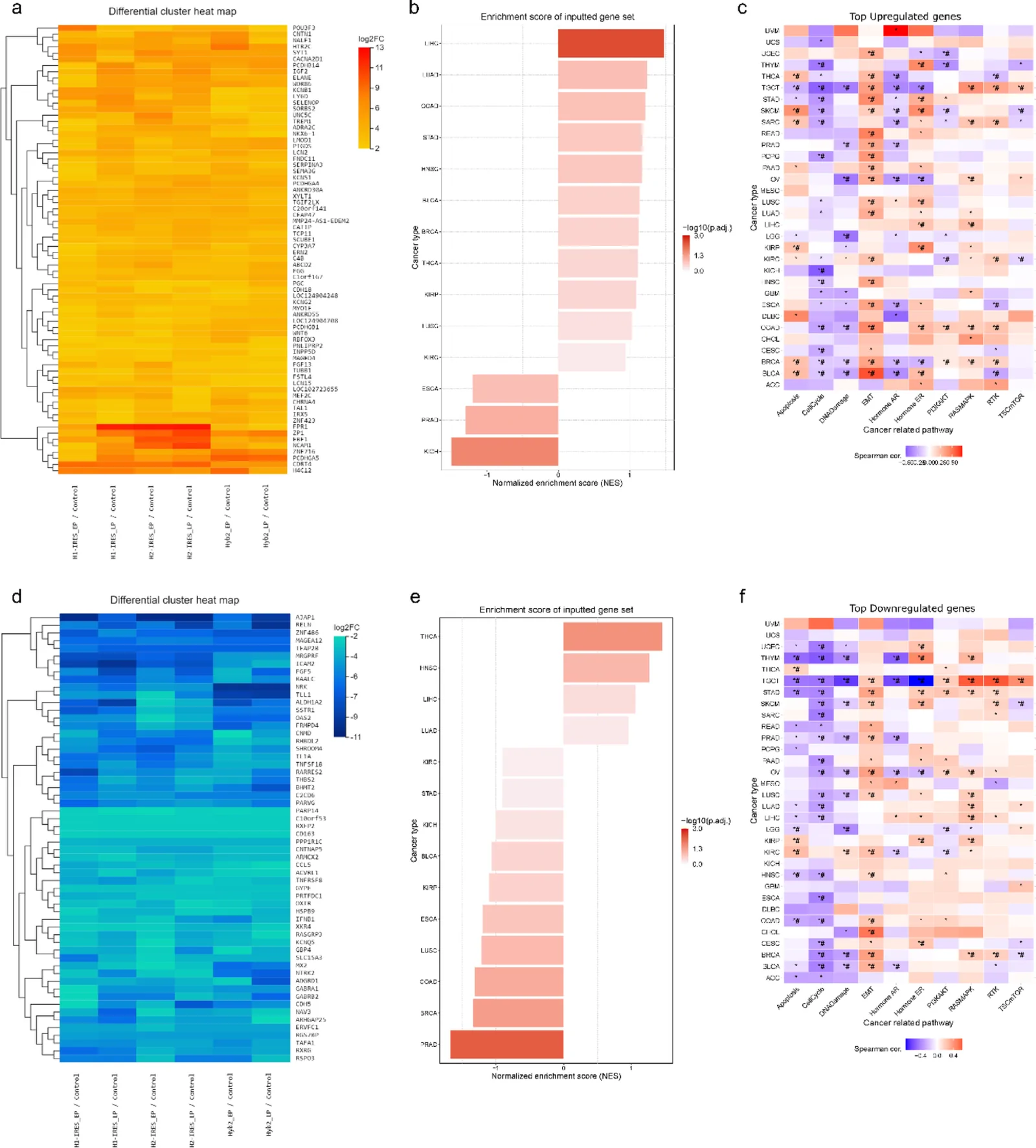
The top up- and downregulated genes of the hybrids correlated with cancer-related genes and pathways. (**A**) Commonly highly upregulated genes (log2FC > 2) in early- and late-passage hybrids compared with M13SV1 control cells, depicted as a heatmap. (**B**) GSEA enrichment analysis of the top upregulated genes in hybrid cells compared with the 20,000 + gene set from the GSCA database. (**C**) The correlation matrix indicates whether the upregulated genes of the hybrids correlated with the activation or inhibition of certain cancer-related pathways in diverse cancer types. (**D**) Most common downregulated genes in hybrids (log2FC < -2) and the (**E**) NES calculation from the GSCA database. (**F**) Correlation analysis of the top downregulated genes among the hybrids with cancer-related pathways. Most of the genes whose expression was upregulated or downregulated in the hybrids correlated with EMT activation, whereas the cell cycle pathway seemed to be inhibited in most cancer types. All data represent three independent measurements. Spearman correlation with * *P* value < 0.05; #FDR < 0.05

To identify consensus differentially expressed genes (DEGs) across the hybrid clones, we evaluated the transcriptomic profiles using the thresholds q ≤ 0.05 and |log2FC| ≥ 0.5. The UpSetR plot revealed that 21 DEGs were expressed only in early-passage hybrid clones, whereas 132 DEGs were expressed only in late-passage hybrid clones (Fig. 7a; Supplementary File S4a-e). Compared with the parental cells, all hybrid clone cells expressed 427 identical DEGs, which were used for downstream studies. First, the common DEGs of the hybrids were subjected to enrichment analysis via MiSigDB (Fig. 7b). The top enriched terms for the input gene set were genes related to the response to IFN-α and IFN-γ, EMT, hypoxia or p53 signaling (Fig. 7b). The 427 aberrantly expressed genes common to all hybrid clone cells were plotted according to their q-values and log2FC values to identify which genes were significantly up- or downregulated across hybrid clones and passages. For subsequent analyses, the genes *IGF2*, *MAGED4*, *EYA4* and *TLL1* were selected as those genes that were significantly higher ranked in late-passaged cells and their cancer-association scores were determined using the citation-based OncoScore prediction tool (Fig. 7d). When addressing the score rank, *MAGED4* is listed as the highest potential oncogene with a citation-based OncoScore of over 70, followed by *IGF2* (score = 40), *EYA4* (score = 35), and *TLL1* (score = 15). Network interaction analysis of several important terms was performed to identify the hub genes of these particular clusters. In the EMT network, high connectivity was detected for *FGF2* and *THY1*, while the highest degree of connection in p53 signaling and hypoxia was detected for *CDKN1A* (Fig. 7e). We further analyzed the most abundant (log2FC > 2) and downregulated genes (log2FC < -2) of the hybrid clones. Overall, 72 genes were highly upregulated in the hybrid clones compared with a total of 58 downregulated genes (Fig. 8a + d). To address the query of whether the differentially expressed genes of the hybrid clones correlated with the gene expression of certain cancer types, we transferred these data into the GSCA pipeline and scrutinized whether the input gene sets were over- or underrepresented in some cancer types. By plotting the hybrid clone gene sets against 20,000 + genes from the GSCA database, we observed that most genes that were upregulated in the hybrids were also upregulated in many diverse cancer types (Fig. 8b). Similarly, genes that were commonly downregulated in the hybrid clones were predominantly underrepresented in several distinct cancer types (Fig. 8e). We also used the given hybrid clone gene set and investigated whether it correlated with the activation or inhibition of certain cancer-related pathways provided by the GSCA server. Correlation analysis using the GSVA score module showed that the up- and downregulated hybrid clone gene sets correlated with the activation of the EMT pathway in a broad range of cancer types, whereas the cell cycle in many cancers was suppressed mainly by the implemented hybrid clone gene set (Fig. 8c + f). These findings indicate that the transcriptomic signatures of M13-IRES-Hybrids-1 and -2, and M13-Hybrid2 cells share similarities with the gene expression profile of various cancer types.

### Hybrid formation is associated with the transcriptional downregulation of the p36 locus on chromosome 1

**Fig. 9:**
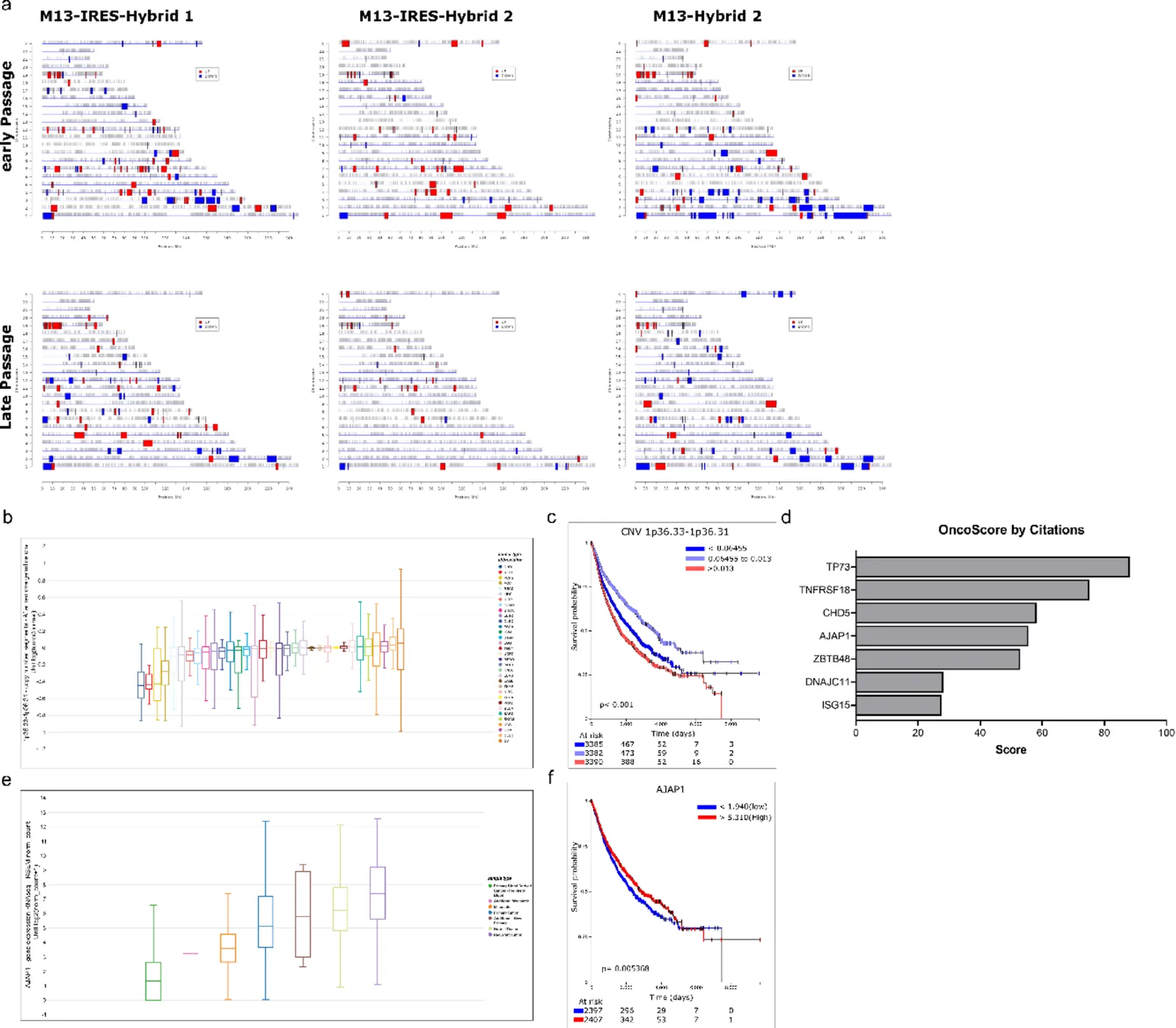
Identification of transcriptional highly significant up- or downregulated chromosomal regions in the hybrid genome. (**A**) PREDA software was used to monitor transcriptionally significantly up- or downregulated genes across the hybrid genome, which may indicate putative gains and losses of whole chromosomes/arms. An FDR threshold of 0.01 was selected for statistical significance. Strikingly, the chromosomal region p36 on chromosome 1 seemed to be significantly downregulated in all the hybrids, suggesting transcriptional silencing or a putative deletion of this chromosomal arm. (**B**) Copy number variation (CNV) of 1p36.33-1p36.31 across diverse cancer types. The TCGA Pan-Cancer dataset (*n* = 12,839) was used to explore pan-cancer associations and to visualize copy number loss (CNL) and copy number gain (CNG) of 1p36.33-1p36.31. Notably, many cancers exhibit a CNL rather than a CNG of this particular chromosomal region. One-way ANOVA was used (*P* < 0.001, f = 43.72). (**C**) Kaplan‒Meier overall survival plots of cancer patients with a CNL of 1p36.33-p36.31 from the TGCA database. Analysis of the top, middle and bottom 33% of CNVs of 1p36.33-1p36.31. High copy number loss and a gain of 1p36.33-1p36.31 are linked with an overall worse survival outcome. (**D**) Predictive analysis of the putative cancer-associated potential for genes according to the literature-based prioritization tool OncoScore in the 1p36.33–1p36.31 region that are significantly downregulated across all hybrids. (**E**). Box plot diagram of *AJAP1* expression between the different cancer subgroups and normal tissues obtained from the cohort studies of the TCGA, TARGET and GTEx datasets (*n* = 17,221) from the UCSC Xena platform. The data revealed that especially in metastatic wounds and in the circulation system, cancer cells exhibited significantly lower expression levels of *AJAP1*. One-way ANOVA was used (*P* < 0.001, f = 204.5). (**F**) Survival TGCA cohort study of patients with high or low expression of *AJAP1*. The survival rates of the top 25% and bottom 25% of patients expressing *AJAP1* were analyzed, and demonstrated that lower expression of *AJAP1* was significantly associated with a higher mortality rate in cancer patients.

Chromosomal mapping of the transcriptional regions that were significantly up- or downregulated in the hybrid clones revealed a heterogeneous distribution of over- and underexpressed regions that varied among the hybrid clones as well as across the passages (Fig. 9a). Interestingly, the chromosomal region p36 on chromosome 1 was significantly down-regulated, whereas the p13 locus located on chromosome 19 was significantly up-regulated in all hybrid clones (Fig. 9a). Whether these findings were related to possible copy number gains or losses in these regions or to gains and losses of whole chromosomes or due to other mechanisms remain unclear. However, additional analysis unveiled that only 1p36 encompassed a common set of genes whose expression was downregulated in all hybrid clones, while no identical, overlapping region was found for the 19p13 chromosome (Fig. 9a). Since 1p36 is a hotspot of several tumor suppressor genes (TSGs), we first consulted the TSGene database to screen for tumor suppressor genes located at 1p36. A hit rate of 31 TSGs was found and used to analyze the differences among the groups via the log2-fold-change filter (Supplementary Table. S2). As expected, most of the TSGs were predominantly downregulated in the hybrid clones with respect to the parental M13SV1 cells. Thereby, *AJAP1* was the most downregulated TSG with a negative log2FC value ranging from -6 to -10 among the hybrid clones. Further significantly downregulated TSGs in all hybrid clones were *ISG15* and *TP73*, while *TNFRSF18* and *CAMK2N1* were only markedly downregulated in the M13-Hybrid2 cells. Since the most highly downregulated TSGs span the p36.33-p36.31 region on chromosome 1, we utilized this information to delve deeper into whether the loss of this specific region potentiates the likelihood of tumorigenesis. In fact, deletion of 1p36 has been reported to be a common phenomenon in various cancer types [45]. The USCS xena platform provided to explore copy number variations across the TCGA Pan-dataset revealed that the chromosomal region 1p36.33-1p36.31 is frequently deleted in many cancers, such as cholangiocarcinoma, pheochromocytoma or renal cancer (Fig. 9b), and is further related to a worse prognosis for cancer patients suffering from this chromosomal deletion (Fig. 9c). Gene-ranking related to cancer-association further revealed a high-ranked citation-based OncoScore for all detected downregulated genes that span the 1p36.33-1p36.31 region ranging from 30 to approximately 90 (Fig. 9d). Moreover, *AJAP1* gene expression was more downregulated in cancer tissue than in normal healthy tissue, and its expression was even more reduced in metastatic lesions, suggesting that *AJAP1* downregulation might be a prognostic marker for cancer development (Fig. 9e). Similarity, exploratory Kaplan–Meier survival analysis of the pan-cancer dataset indicated that *AJAP1* downregulation was correlated with poorer overall survival of cancer patients (Fig. 9f)

### The colony- and mammosphere-formation capacity was enhanced in the hybrid clones

**Fig. 10:**
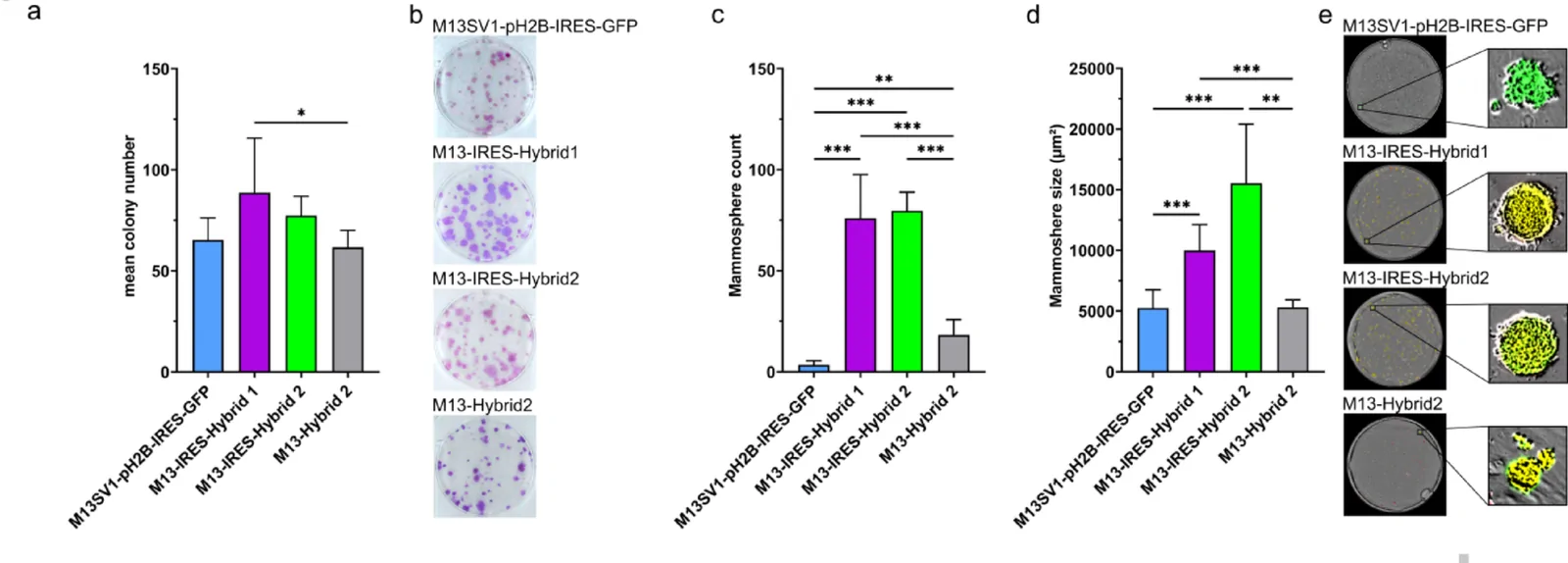
Hybrid cells exhibit significantly larger colony and mammosphere formation capacities than the parental cells. (**A**) Hybrids formed more colonies than diploid M13SV1 parental cells. A one-way ANOVA test coupled with a Tukey’s post hoc test were used (* *P* < 0.05). (**B**) Shown are representative images of colonies derived from the indicated cell lines. (**C**-**D**) Mammosphere formation and size revealed hybrid-clone-dependent differences, whereby compared with the M13-Hybrid2 clone, the M13-IRES-Hybrid1 and M13-IRES-Hybrid2 clones generated an overall higher number of mammospheres and considerably larger mammospheres. Data were analyzed using a one-way Welch’s ANOVA, followed by a Dunnett’s T3 post hoc test (**E**) Representative Images of fluorescent mammospheres from the indicated cell lines

Phenotypic studies were conducted to analyze the self-renewal potential of the hybrid clones. We therefore used hybrid clones from intermediate and late passages (P. 15-P. 30) to explore their ability to form colonies and mammospheres (Fig. 10a‒e). The colony formation assay demonstrated that the ability to form colonies varied among the individual hybrid clones. In fact, M13-IRES-Hybrid1 cells had the highest colony-formation capacity, closely followed by M13-IRES-Hybrid2 cells, with a marginally lower number of colonies (Fig. 10a). The M13-Hybrid2 cells revealed the lowest colony-formation capacity, which was comparable to the colony formation capacity of parental M13SV1 cells. The mammosphere formation capacity of the hybrid clones was subsequently examined (Fig. 10c). Consistent with the results of the colony formation assay, the M13-IRES-Hybrids also exhibited a significantly increased ability to form mammospheres, with both hybrids also producing the largest mammospheres (Fig. 10c-d). Of note, the largest mammospheres were generated by M13-IRES-Hybrid2 cells, which were approximately 15,000 µm^2^ in size, while the average size of M13-IRES-Hybrid1 mammospheres was about 10,000 µm^2^ (Fig. 10d). In contrast, the mammosphere formation capacity of M13-Hybrid2 cells was significantly lower compared to M13-IRES-Hybrids (Fig. 10c). Similarly, M13-Hybrid2 cells formed rather small mammospheres with an average size of 5,000 µm^2^ in size, which was comparable to parental M13SV1 cells (Fig. 10d). Taken together, these results show that M13 hybrid cells generated by homotypic cell fusion exhibited an increased colony and mammosphere formation capacity, which might be related to putatively enhanced stemness traits.

### Hybrid cells revealed differences in invasive capacity, reflecting their specific EMT phenotype

**Fig. 11:**
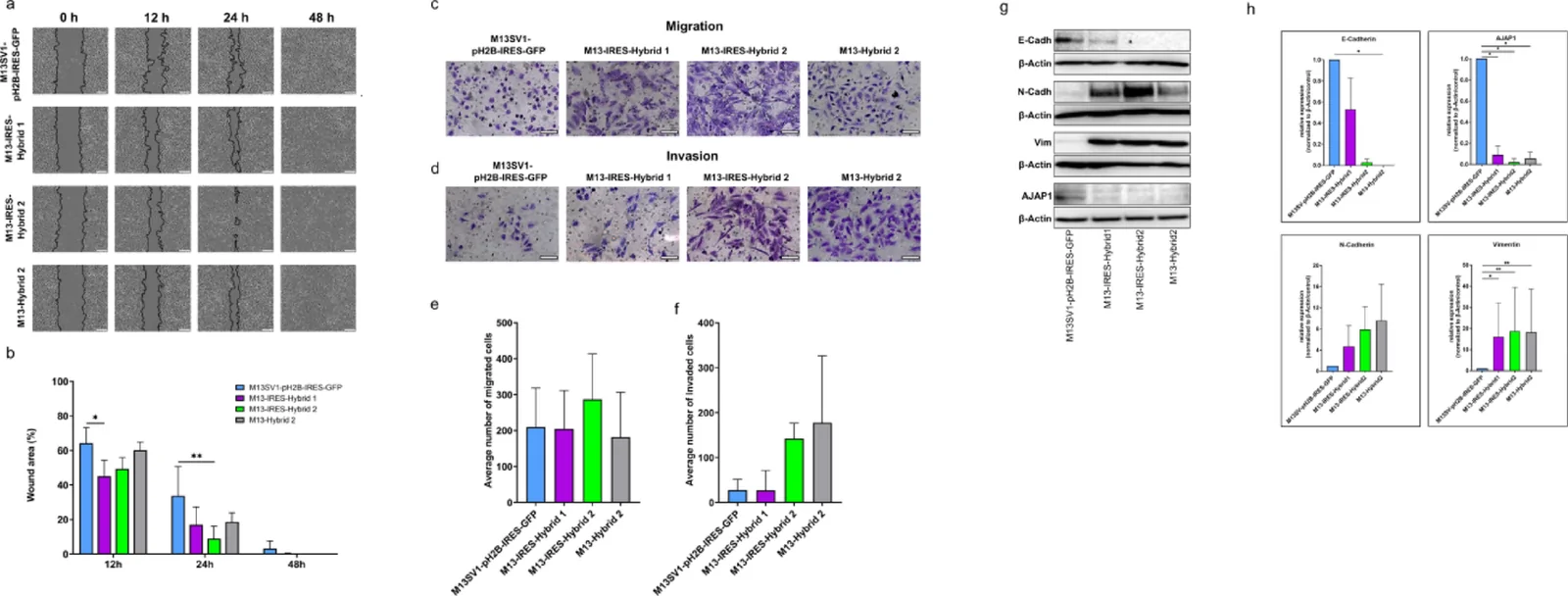
Hybrid cells undergo EMT and exhibit clone-dependent migratory and invasive capabilities. (**A**) Cell migration was analyzed by measuring the wound width after scratching. Here, the percentage of the remaining cell-free area was determined at time intervals of 0 h, 12 h, 24 h and 48 h (scale bar = 200 μm). (**B**) Quantitative evaluation of the results of the wound-healing assay. A two-way ANOVA for three independent experiments and a Tukey post hoc test were used for wound scratch validation (* *P* < 0.05, ** *P* < 0.01). (**C**-**D**) Representative images of the Transwell migration and invasion assays, respectively, for the indicated cells (scale bar = 100 μm). (**E**-**F**) Evaluation of the respective hybrid and parental cells revealed hybrid-clone-dependent discrepancies in migratory and invasive capacities. Migration and invasion assays were analyzed using a one-way ANOVA with a Tukey post hoc test; no significant differences were observed among the groups (*P* > 0.05). (**G**) Western blot data of EMT marker expression indicate that hybrid cells have undergone EMT. (**H**) Densitometric validation for the three Western blots was normalized to β-actin and the M13SV1-pH2B-IRES-GFP control group. Data analysis used β-actin-normalized data to ensure variance homogeneity, and statistical analysis was performed independently for each target protein based on data distribution. A Kruskal-Wallis test followed by Dunn’s post hoc test was applied for E-cadherin, while a one-way ANOVA followed by Tukey’s post hoc test was used for the other targets (* *P* < 0.05, ** *P* < 0.01). Interestingly, the M13-IRES-Hybrid1 clone expressed both E-Cadherin and N-Cadherin, suggesting a more intermediate EMT phenotype. Shown are representative data of three independent measurements

To assess the migratory behavior of hybrid clones, a wound-scratch assay and a transwell migration assay were conducted. Wound-scratch closure was monitored over the indicated time intervals (Fig. 11a-b) and revealed an overall higher migratory potential of all hybrid clones compared to M13SV1 parental cells. Thereby, M13-IRES-Hybrid2 cells possessed the highest migratory activity of all hybrid clones (Fig. 11a, b). In accordance to Wound-scratch assay data, we also observed that M13-IRES-Hybrid2 cells possessed the highest migratory activity in a transwell migration assay (Fig. 11c, e). Interestingly, the transmigratory activities of M13-IRES-Hybrid1 cells and M13-Hybrid2 cells were similar to that of M13SV1 parental cells (Fig. 11c, e). In addition to cell migration, we also investigated the cells’ invasion capacities (Fig. 11d, f). Here, M13-IRES-Hybrid2 and M13-Hybrid2 cells exhibited the highest invasive potential of all cell lines. Although this was not statistically significant, the number of invaded M13-IRES-Hybrid2 and M13-Hybrid2 cells was 5- to 10-fold higher as compared to M13-IRES-Hybrid1 cells and M13SV1 parental cells (Fig. 11d, f). Since epithelial‒mesenchymal transition is closely intertwined with cell motility, we explored whether hybrid clones may have undergone EMT and acquired a mesenchymal-like phenotype. Western blots were performed to determine the expression of classical EMT markers, such as E-/N-Cadherin and Vimentin (Fig. 11g). M13SV1 cells exhibit a classical epithelial phenotype, which is in agreement with published data [46] The cells are positive for E-Cadherin, but negative for N-Cadherin and Vimentin expression (Fig. 11g). In contrast, N-Cadherin and Vimentin expression was detected in all hybrid clones (Fig. 11g), which indicate that the cells likely have undergone EMT. Interestingly, E-Cadherin expression was only fully down-regulated in M13-IRES-Hybrid2 and M13-Hybrid2 cells (Fig. 11g). In contrast, slightly lower E-Cadherin expression levels were still detectable in M13-IRES-Hybrid1 cells, which may indicate that the cells have undergone a partial EMT (Fig. 11g). It is known that cancer cells can adopt a so-called intermediate or mixed E/M state, which is not only characterized by the co-expression of epithelial and mesenchymal markers, but also by cancer stem cell-like properties [47]. However, the co-expression of E-Cadherin, N-Cadherin and Vimentin in M13-IRES-Hybrid1 cells is not sufficient to conclude that the cells have adopted this so-called intermediate or mixed E/M state. Additional in vitro and in vivo studies are recommended. We also examined the expression of the adherens junction associated protein 1 (AJAP1), a well-known epithelial cell marker and one of the top downregulated genes in all hybrid clones and M13SV1 parental cells (see Fig. 7c, Supplementary Table. S2). In accordance with the transcriptomic analysis, AJAP1 was detected in M13SV1 cells, but its expression was almost completely abrogated in all hybrid clones (Fig. 11g, h).

Similarly, the expression of E-cadherin, N-cadherin, and vimentin in the hybrid cells likely appears to correlate with the invasion data (Fig. 11d, f, g). M13-IRES-Hybrid2 and M13-Hybrid2 cells express only N-cadherin and vimentin, and both clones exhibit increased invasive potential. In contrast, M13-IRES-Hybrid1 cells express all of the EMT markers examined but are not invasive. However, without further studies, it is not possible to conclude that there is a clear relationship here. Nevertheless, these findings indicate that epithelial cell fusion can generate hybrid clone cells with a motile mesenchymal phenotype. However, transcriptional and in vitro data show that the intensity of this phenotypic switch varies among individual hybrid clones.

## Discussion

Cancer development is a multifaceted process involving sophisticated interactions among genetic, epigenetic, cellular, and environmental mechanisms that drive the onset and progression of cancer [48]. Consequently, cancer is underpinned by a dynamic evolutionary process resulting in the selection of subclones with survival benefits that outcompete and displace less competitive players and enable rapid adaptation to stressors and environmental changes [49]. The etiology of cancer involves numerous hypotheses that postulate how malignant transformation can occur and can contribute to the development of cancer, such as the classical evolution model, the cancer stem cell hypothesis, or a single, decisive catastrophic event that ultimately provokes a malignant phenotype [50–52]. In fact, single catastrophic events in cancer have been shown to be more common than first assumed, often causing massive, multi-genomic changes at once, culminating in a genome crisis known as chromothripsis or chromoplexy. Pan-cancer analysis of over 2,600 tumors across 38 cancer types revealed 22.3% chromothripsis and 17.8% chromoplexy incidence, both occurring early in cancer evolution and driving massive chromosomal rearrangements and aneuploidy [53]. Therefore, single or few catastrophic events can drastically accelerate cancer evolution, whereby multiple malignant traits can be acquired in a single stroke. Atypical cell fusion may also represent a type of catastrophic cellular event in which the newly fusion-derived hybrid cell instantly has to cope with a doubling in genome size. During the first mitosis, when the parental genomes fuse together, genome chaos ensues, which is further fueled by chromosomal and mitotic aberrations, ultimately promoting the expansion of genetically deviant hybrid progeny. This study elucidated how aberrant cell fusion events in non-cancerous cells may serve as an initial catastrophic mechanism to instigate the devastating route to malignancy. For this purpose, we analyzed hybrid cells generated via homotypic cell fusion using the non-transformed M13SV1 breast epithelial cell line and traced three randomly selected hybrid clones (M13-IRES-Hybrid1, M13-IRES-Hybrid2 and M13-Hybrid2) from early to late passages to explore the karyotypic, morphometric, and transcriptomic evolution over time.

Karyotype analysis revealed a hybrid clone-dependent ploidy reduction process across passages, shifting from an initial near-tetraploid state to a near-diploid or near-triploid state. Strikingly, the number of mitotic errors remained constant (Fig. 3a), indicating that ploidy reduction was not primarily related to mitotic fidelity. Instead, CIN and DNA damage remained persistently high throughout the passages, suggesting that hybrid cells experienced ploidy-dependent replication stress. This stress likely arose from nonproportional scaling of nucleosides/replication factors or stoichiometric imbalances that disrupt cell cycle regulators, causing over- or under-replicated DNA regions and strand breaks [25, 26, 54]. Furthermore, replication stress acts as a key driver, triggering *de novo* structural aberrations that lead to mitotic segregation errors, while additionally contributing to numerical, whole-chromosome changes in progeny cells [16, 19]. To survive, polyploid and aneuploid cells overcome replication stress-induced arrest by upregulating DNA repair genes and activating error-prone non-homologous end-joining (NHEJ), which inherently promotes CIN [55, 56]. This finding is in line with our data, showing a high degree of DNA damage during S phase that carried over into M phase. Despite the transcriptional upregulation of DNA repair genes in the hybrids (Fig. 6c), improper repair likely promoted subsequent mitotic chromosomal aberrations.

Notably, hybrid clone cells presented an overall increased survival rate compared to diploid M13SV1 cells, where abnormal mitoses frequently resulted in the purging of progeny. It might be speculated that diploid M13SV1 parental cells are more susceptible to the Muller’s ratchet effect, where daughter cells suffer from lethal gene losses as a consequence of chromosome missegregation driven by mitotic aberrations, such as multipolar cell division [57]. Since hybrid cells typically bear more than two copies of each chromosome, they might be more resilient against homozygous loss of essential genes. We recorded a range of diverse mitotic anomalies in all hybrid clones throughout the passages, including multipolar cell division, endomitosis and lagging chromosomes (Fig. 3a, b, Supplementary movies S1-S18). Unlike most other WGD events, cell fusion leads to cytoplasmic exchange and centrosome overduplication, making cells highly vulnerable to multipolar mitoses and segregation defects. While hybrid clone cells exhibited a higher occurrence of multipolar mitoses, their micronuclei formation levels were not proportionally higher than those of the M13SV1 parental cells. This finding is interesting since tetraploid cancer cells typically form more micronuclei than their diploid counterparts [16, 58]. Nevertheless, the size threshold filter utilized may have resulted in inaccurate scoring. Specifically, the area filter based on common criteria [59, 60], which requires micronuclei to be < 1/5 the size of the primary nucleus, likely excluded larger micronuclei, thereby skewing the counts. Interestingly, we found that early-passage hybrid clone cells formed larger micronuclei on average than late-passage hybrid clone cells, suggesting a link between ploidy level and the capacity to form larger micronuclei. The causes of micronuclei formation can differ, including chromosome misalignment [61], chromosome breakage with the emergence of acentric or dicentric fragments [62, 63], and nuclear/anaphase bridges or lagging chromosomes [22, 64, 65], all of which decisively dictate micronucleus size. For instance, DNA double-strand breaks generally cause smaller micronuclei than displaced or lagging chromosomes. Our results showed that hybrid clones harbored micronuclei of heterogeneous sizes that were also clustered around the main nucleus, which is a common phenomenon in PGCCs [66, 67]. Chromosome content analysis has shown that these micronuclei can vary tremendously in their chromosomal material [68]. Despite the lack of data, WGD and, in general, polyploidy may result in larger micronuclei simply attributed to the larger chromosomal constitution or extra centrosomes.

By contrast, CIN, or more broadly, genomic instability, can drive endoreplication, either by disrupting cell cycle checkpoints or through mitotic segregation errors [54, 69–71]. For instance, oncogene-induced replication stress via elevated cyclin E levels promoted WGD in p53-proficient cells by suppressing mitotic CDK activity, thereby triggering mitotic bypass [54]. However, p53 deficiency is widely recognized as a prerequisite for cells to bypass the G1/S checkpoint, undergo WGD, and proliferate despite DNA damage [72, 73]. In addition, overexpression of cyclin D or E can overcome p53-dependent cell cycle arrest, enabling WGD and the continued proliferation of tetraploid cells [74, 75] Concurrently, other p53 family members, such as p63 and p73, can compensate for the loss of p53 function [76], and the codeletion of p53 and p73 increases the propensity of cancer cells for continued endoreplication and polyploidization [77]. Under this aspect, p73 plays a crucial role in genome stability by activating the mitotic checkpoint and safeguarding against p53/p73-induced apoptosis [78, 79]. Our data revealed compromised expression in two of the three hybrid clones, suggesting a putative p53 deficiency corroborated by Western blot analyses (Supplementary Fig. S5a-b). Similarly, a significant downregulation of p73, combined with p53 loss in hybrids, likely sustained proliferation despite elevated DNA damage and polyploidy. Intriguingly, live-cell imaging revealed that incomplete or failed division triggered the formation of giant, mono- or multinucleated hybrid cells (Figs. 3b and 4a) still capable of proliferating via multipolar cell division or additional endoreplication. While such cell cycle re-entry is highly atypical for normal human cells [80], it is strongly associated with genomic instability and oncogenesis [18, 37, 54]. One of the most fascinating findings was that some hybrid cells were capable of refusing with each other to undergo erroneous mitosis, which still remains ambiguous (Fig. 3a-b, Supplementary movies. S2-S18). A similar observation was reported in Hodgkin lymphoma cells, where mononucleated Reed–Sternberg cells continuously refuse with one another to form large multinucleated cells, a process exclusively undertaken among daughter cells following mitosis [81].

Cell size scaling revealed hybrid- and passage-dependent heterogeneity, whereby cell size scaled proportionally with nuclear size (Fig. 4b, Supplementary Fig. S1). Average values showed that both cell and nuclear sizes decreased after several passages, consistent with previous findings on ploidy reduction. Robust cell-to-nuclear linearity is a fundamental biological aspect and emphasizes the importance of fulfilling cellular demands, which otherwise can result in cellular dysfunction associated with pathological outcomes [82–84]. Under most physiological conditions, ploidy levels are tightly interwoven to cell size, and increasing DNA content typically accompanies with cell enlargement [85, 86]. On the other hand, cell size is also concurrently regulated by non-genomic mechanisms, such as mechanical and environmental factors, cytoplasmic composition, nucleocytoplasmic transport, and transcriptional machinery [87]. However, the nuclear‒cytoplasmic (N/C) ratio has become an established scaling criterion for pathologists to distinguish healthy from malignant cells since, in cancer cells, N/C ratio is often unbalanced [84, 88, 89]. In our study, no significant changes in the N/C ratio were observed in early-passage hybrid clones vs. parental M13SV1 cells. However, late-passage M13-Hybrid2 cells exhibited a more aberrant N/C ratio. Although nuclear size increased in proportion to cell size, no strict linear correlation between WGD and cell enlargement was observed. Yet, the relationship between cell size and ploidy remains controversial, and even if, in some eukaryotes, cell size is proportional to genome size, this is not a universal feature across species [82, 90]. ]. Additionally, altered cell size or N/C ratios can disrupt spindle architecture [91–93] and delay SAC signaling, triggering mitotic bypass and chromosomal missegregation. Although larger cells may exhibit a weaker SAC response [94, 95], factors such as spindle pole size and microtubule abundance are more critical for mitotic fidelity; thus, the N/C ratio alone is insufficient to evaluate malignancy [92–94]. Nevertheless, early-passage hybrid clones exhibited a significant downregulation of spindle assembly gene expression (Fig. 6c), suggesting that compromised spindle dynamics may cause mitotic arrest and reduced proliferation capacity.

Finally, nuclear atypia was examined as a further indicator of cellular premalignancy. Nuclear deformation, a hallmark of cancer cells [96], suggests that nuclear envelope defects can trigger deformation-induced DNA damage [97], CIN [98] or inflammation [99]. Nuclear deformation was markedly elevated in all hybrid clones and did not ameliorate in later passages (Fig. 5a-b). Correlation analysis demonstrated that DNA damage was strongly correlated with nuclear dysmorphia, particularly in M13-Hybrid2 cells (Fig. 5c). Although nuclear deformation is not the sole mechanism of DNA damage, the mutual reinforcement of CIN and nuclear rupture drives nuclear lobulation and folding, supporting our findings of a constantly high CIN within the hybrid clones.

Since hybrid clones exhibited elevated CIN levels with hybrid- and passage-dependent karyotypic and morphological differences, we sought to detect transcriptome-wide discrepancies in a putative hybrid-specific and temporally regulated expression pattern. Scaling of the gene expression profile clearly revealed that hybrid clones presented a differential gene expression profile (Fig. 6a-b). Similarly, gene clustering unveiled an upregulation of genes associated with EMT, locomotion and cell motility. It is well-known that the fusion of cancer cells with somatic cells, such as macrophages and stem cells, can give rise to tumor hybrids that exhibit novel properties, such as an enhanced drug resistance, an increased metastatic behavior (and thus EMT) and even cancer stem cell-like properties [100–102]. Current research indicates that fusion between mesenchymal stem/stromal cells and epithelial cells can induce EMT in hybrid cells, thereby promoting invasiveness and tumor formation [103]. Yet other studies have shown the exact opposite effect, where cell fusion between MSCs or BMDCs and epithelial cells is more likely involved in epithelial transformation [104, 105]. Here, we demonstrate, for the first time, that atypical spontaneous cell fusion among breast epithelial cells can result in hybrid clone cells with an EMT-like phenotype. Interestingly, as shown here, all hybrid clones possessed an altered EMT score profile, whereby substantially greater EMT scores were observed in late-passage hybrid clones as compared to early-passage hybrid clones. Moreover, GSEA indicated altered EMT progression profile among the hybrid clones, whereby not all clones underwent EMT during early passages, suggesting that EMT is a subclonal evolutionary process rather than an immediate consequence of fusion. Additionally, GSEA suggests that hybrid clones develop a more stable mesenchymal phenotype over time. Whether this is due to subclonal evolution, or even occurs during PHSP, remains to be clarified in subsequent studies. Overall, the gene expression and EMT score data are in good agreement with Western blot data and partially in agreement with migration and invasion data. As shown, M13-IRES-Hybrid2 and M13-Hybrid2 hybrids were negative for E-Cadherin, but positive for N-Cadherin and Vimentin. Similarly, both hybrid clones possessed a markedly enhanced invasion capacity suggesting that both hybrid clones may have undergone EMT. However, further studies are mandatory, such as expression analysis of additional epithelial and mesenchymal markers, to conclude whether M13-IRES-Hybrid2 and M13-Hybrid2 hybrids have truly undergone a full EMT. This applies as well to M13-IRES-Hybrid1 cells. Western blot analysis revealed a co-expression of epithelial (E-Cadherin) and mesenchymal markers (N-Cadherin and Vimentin), which likely indicate that the cells may have acquired a so-called intermediate or mixed E/M state [47, 106]. Cancer cells in an intermediate or mixed E/M state exhibit a high tumor-initiation capacity, suggesting that they possess cancer stem cell-like characteristics. Thus, the finding that M13-IRES-Hybrid1 cells exhibit both an enhanced colony formation and mammosphere formation capacity may support the assumption that the cells might have acquired an intermediate or mixed E/M state. However, M13-IRES-Hybrid2 cells also exhibited an enhanced colony formation and mammosphere formation capacity, which likely indicate putative cancer stemness traits, but lack E-Cadherin expression, and, hence, might not be in an intermediate or mixed E/M state. Therefore, additional in vitro and in vivo studies are recommended for further characterization of the hybrid clones. Without limited-dilution assays and serial transplantation experiments, it is impossible to determine whether hybrid clones possess the ability to induce tumors and exhibit cancer stem cell properties.

While the upregulation of hypoxia- and glycolysis-related genes often occurs in polyploid cells to fulfill the metabolic demand of increased energy supply [107], the overexpression of xenobiotic genes, including *ABCD2*, *PDLIM5* or *HMOX1*, might significantly contribute to drug resistance among hybrid clones [108–110]. As addressed above, hybrid clones displayed a high upregulation of genes linked with p53 signaling, such as *CDKN1A*/p21 and *CCND2*, suggesting that hybrid clones may overcome p53/p21 cell cycle arrest through the overexpression of cyclin D2 [74]. Abundant *CDKN1A*/p21 expression without sustained *TP53* expression implicates a p53-independent p21 activation (Supplementary Fig. 5a-c), which has been reported to be a major contributor to genomic instability, chemotherapeutic resistance, cell motility and metastasis [111–113]. A more recent study demonstrated that p21 is a hub regulator of PGCC formation; thus, the inhibition of p21 expression might be a promising target for preventing PGCC formation and progeny generation [114]. We identified *CDKN1A*/p21 as a central core gene within the p53 and hypoxia network among the hybrids, suggesting that *CDKN1A*/p21 might be a key regulator in both networks. Hence, targeting *CDKN1A*/p21 might represent a promising strategy to interrupt potential cross-interactions, abrogate hypoxia-induced metabolic changes and promote hybrid cell death. Conversely, significantly downregulated DEGs were enriched in inflammatory pathways required for immune cell activation and pathogen/tumor cell clearance (Fig. 7e, Supplementary Fig. S4) [115, 116]. In this context, a diminished IFN-α/γ response could facilitate immune evasion, in which downregulating interferon-stimulated genes (ISGs) may dampen immune infiltration to sustain hybrid survival. However, the role of ISGs is multifaceted, and their role in tumor promotion or suppression depends heavily on the specific ISGs involved, the cell type, and the surrounding microenvironment, necessitating further investigation through in vivo studies [115].

Transcriptional chromosomal mapping of significantly up- and downregulated regions pinpointed 1p36 as a locus with commonly downregulated genes in all hybrid clones (Fig. 9a, Supplementary Tab. S2). This finding is particularly noteworthy since the loss of 1p36 is a common event in various cancers [45], and in-depth analysis revealed 1p36.33-1p36.31 as the region where TSGs were significantly downregulated (Supplementary Tab. S2). Within this region, *AJAP1* was the most prominently downregulated gene and Western blot analysis confirmed the complete absence of AJAP1 expression in all hybrid clones (Fig. 11g). Notably, AJAP1 has been suggested as an inhibitor of EMT [117, 118]. For example, overexpression of AJAP1 inhibited EMT, migration, and invasion of prostate cancer cells, while silencing or increased methylation of AJAP1 had the opposite functions [118]. In this regard, it would be interesting to investigate in ongoing research whether the downregulation of AJAP1 expression was correlated with the putative EMT phenotype in the hybrid clones. By doing so, it would also be interesting to study the cause of AJAP1 downregulation in hybrid clones. Even if the 1p36 region is not entirely deleted in hybrids, AJAP1 downregulation can be attributed to partial inactivation of the *AJAP1* gene by copy number loss, miRNA regulation, promotor hypermethylation or posttranscriptional degradation [45].

## Conclusion

Overall, in this study, we systematically classified three individual hybrid clones originating from homotypic cell fusion of non-transformed breast epithelial cells according to their karyotypic, morphological, transcriptomic and phenotypic properties and how these features have changed as a consequence of a dynamic evolutionary selection process. We demonstrated that hybrid clones were extremely CIN positive across passages, as illustrated by the high number of mitotic aberrations and the high incidence of cell and nuclear pleomorphism. Trajectories of the hybrid clones during long-term cultivation revealed that some hybrid clones have developed a PGCC-like phenotype, which is triggered by continuous endoreplication, cell fusion, or cytokinetic failure. Transcriptional validation revealed a widespread downregulation of 1p36-localized genes. This region is a well-known locus often deleted across many cancer types and known to drive malignancy. Here, the 1p36 located gene *AJAP1* was the most heavily downregulated gene in all hybrid clones, which suited well to the hybrid clones’ putative EMT phenotype. Further characterization of hybrid clones regarding, colony- and sphere-forming ability, and migration/invasion capacity support the highly premalignant transcriptomic signatures of the hybrid clones. Therefore, aberrant fusion events among non-transformed cells may give rise to hybrid cells that exhibit malignant properties, which may play a role towards a premalignant state and ultimately tumor development.

## Supplementary Information

Supplementary Material 1: Table S1. GSEA of the distinct hybrid clones compared with M13SV1 control cells. Common GSEA data of enriched MiSigDB hallmarks of the hybrid clones unveiled the upregulation of genes associated with EMT, the p53 pathway, hypoxia, glycolysis and drug/xenobiotic metabolism, whereas genes upregulated in response to IFN-α and IFN-γ and genes involved in G2/M progression were downregulated in the hybrids. The table with the NES reveals a different enrichment profile, which was hybrid clone- and passage dependent. The normalized read count of three replicates (*n* = 3; EP = early passage (P.4–6), LP = late passage (P.20-P22)) was selected.

Supplementary Material 2: Table S2. Among the hybrids, *AJAP1* is the most downregulated tumor suppressor gene in the 1p36 region. The expression of 30 tumor suppressor genes (TSGs) localized at 1p36 was predominantly downregulated across the genome of the hybrids. The most significantly downregulated TSG in the hybrids was *AJAP1*, whose log2FC value ranged from -6 to -10.

Supplementary Material 3: Movie S1. 3D time-lapse movie illustrates the appearance of lagging chromosomes in a hybrid cell upon mitosis.

Supplementary Material 4: Movie S2. 3D rotation of a hybrid cell arrested in metaphase, where four spindles indicate tetrapolar mitosis.

Supplementary Material 5: Movie S3. 3D time-lapse movie of a hybrid cell undergoing tripolar mitosis.

Supplementary Material 6: Movie S4. Two hybrid daughter cells fuse together during mitosis.

Supplementary Material 7: Movie S5. Mitotic hybrid cell fuse with interphase hybrid cell and produce multinucleated daughter cell.

Supplementary Material 8: Movie S6. Hybrid cell underwent endocycle and form a big nucleus.

Supplementary Material 9: Movie S7. Hybrid cell underwent endocycle.

Supplementary Material 10: Movie S8. A hybrid cell first undergoes endomitosis and becomes multinucleated after failed mitotic division.

Supplementary Material 11: Movie S9. Endocycle and endomitosis of two hybrid cells.

Supplementary Material 12: Movie S10. Binucleated hybrid cell divide bipolar and form one large multinucleated cell.

Supplementary Material 13: Movie S11: Hybrid cell divide bipolar and generate one multinucleated and one small mononucleated daughter cells.

Supplementary Material 14: Movie S12. Hybrid cell with macronucleus divide to form two multinucleated giant cells.

Supplementary Material 15: Movie S13. Binucleated hybrid cell divide tripolar by which two daughter cells divide normal and one tripolar.

Supplementary Material 16: Movie S14. Multinucleated hybrid cell divides tripolar.

Supplementary Material 17: Movie S15. Two multinucleated hybrid cells fuse together and divide tripolar.

Supplementary Material 18: Movie S16. Binucleated hybrid cell divides tetrapolar.

Supplementary Material 19: Movie S17. Multinucleated hybrid cell divides tetrapolar.

Supplementary Material 20: Movie S18. Hybrid cell divides tetrapolar and form small daughter cells.

Supplementary Material 21: Figure S1. M13SV1 hybrid cells were more enlarged in terms of cell and nuclear size. Notably, hybrids at early passages exhibited an enlargement of the cell and nuclear area during cultivation, which was not observed in late-passage hybrids. In addition, a slight decrease in cell and nuclear size was detected in late-passaged hybrids. To investigate whether the increase in karyoplasm was coupled with cell size growth, the nuclear-to-cytoplasmic ratio was analyzed. Indeed, the increase in the nuclear area was concomitant with the increase in cell size and was minimal compared with the increase in the parental N/C ratio in both early- and late-passage hybrids. All data represent three independent measurements. Two-way ANOVA and Tukey’s post hoc test were used (* *P* < 0.05, ** *P* < 0.01, *** *P* < 0.001).

Supplementary Material 22: Figure S2. Hierarchical enrichment analysis of the 4000 top genes from the K-mean clustering. (**A**-**B**) Enrichment analysis for each cluster depicted as a tree plot, in which the sizes of the dots correspond to the adjusted *P* value and the relationships between the GO terms (**A**) or MSigDB terms (**B**) are displayed via hierarchical clustering.

Supplementary Material 23: Figure S3. Enrichment analysis using different annotation classification methods. The genes with the most significant enrichment of DEGs were annotated using GO biological process (**A**-**B**), KEGG pathway (**C**-**D**) and MiSigDB classification methods (**E**-**F**) and visualized via an enrichment bubble chart (*q* value ≤ 0.05, log2FC ratio ≥ 0.5). A significant enrichment of genes linked to cell migration, EMT and pathways involved in cancer was determined in hybrids. The bubble size represents the number of genes, and the color represents the enrichment significance. Shown are representative data of three independent measurements.

Supplementary Material 24: Figure S4. Identification of hybrid and passage-dependent DEGs. (**A**-**B**) Heatmap of the unique DEGs found in early (**A**) and late-passage hybrids (**B**). The average FPKM of three replicates was used to visualize the mRNA expression of the selected DEGs as a heatmap. (**C**-**E**) The most significant hub genes ranked by the maximal clique centrality (MCC) algorithm from the DEG dataset, including all 427 common DEGs (**C**), 21 genes detected only in early-passage hybrids (**D**) and 132 DEGs detected only in late-passage hybrids (**E**). Shown are representative data of three independent measurements.

Supplementary Material 25: Figure S5. Analysis of TSGs and oncogenes associated with the p53 pathway. (**A**) Gene expression data from the RNA-sequencing study showing genes that were either p53 family members or up- or downstream targets of the p53 signaling axis. A two-way ANOVA and Tukey’s post hoc test were used (* *P* < 0.05, ** *P* < 0.01, *** *P* < 0.001). (**B**) Western blot results for the protein expression of p53, p73, and p21. Shown are representative data of three independent measurements. (**C**) KEGG analysis of p53 signaling visualized with the pathview renderer, exemplified by the M13-IRES-Hybrid1 clone. The fold change (log2FC: -2 to 2) of the DEGs was used to monitor up- and downregulated genes involved in the p53 signaling network of M13-IRES-Hybrid1. The upregulation of MDM2, which serves as a negative regulator of p53, leading to its degradation and downregulation, was detected. However, *CDKN1A*/p21 was markedly upregulated in the hybrids, proposing p53-independent regulation.

Supplementary Material 26: Figure S6. RNA-Seq normalization and transformation pipeline. (**A**) RNA-Seq data were normalized to account for varying sequencing depths by calculating counts per million (CPM). Lowly expressed genes were filtered out by keeping only those with a CPM of 0.5 or higher in at least one sample (**B**-**C**) The normalized data were further transformed using log2 (CPM + 4) to show the distribution of the transformed data as box-plot (**B**) or density plot (**C**). (**D**) The transformed data were then used for PCA and K-means clustering, where the optimal number of groups was determined using the elbow method based on the Within-Groups Sum of Squares metric. A value of k = 4 was chosen as a practical clustering choice for the biological consensus. All data represent three independent measurements.

## Data Availability

The datasets used and/or analyzed during the current study are available from the corresponding author upon reasonable request. The RNA sequencing data used in this study are publicly available and were accessed through the NCBI Gene Expression Omnibus (GEO) under accession no. GSE339186. The normalized read counts used in the iDEP pipeline are provided in the supplementary text file on the GEO database. All the authors have agreed to publish this manuscript. All the authors have agreed to publish this manuscript.

## Abbreviations

BMDCs: Bone marrow-derived dendritic cells
BSA: Bovine Serum Albumin
CIN: Chromosomal instability
CPM: Counts per million
DEGs: Differentially Expressed Genes
DNA: Deoxyribonucleic acid
DSB: DNA strand breaks
EMT: Epithelial–Mesenchymal Transition
FDR: False discovery rate
GO BP: Gene Ontology Biological Process
GSCA: Gene Set Cancer Analysis
GSEA: Gene Set Enrichment Analysis
GSVA: Gene Set Variation Analysis
h: hour
IgG: Immunoglobulin G
IFN-α: Interferon alpha
IFN-γ: Interferon gamma
ISGs: Interferon-stimulated genes
KEGG: Kyoto Encyclopedia of Genes and Genomes
M-phase: Mitotic phase
MET: Mesenchymal-Epithelial Transition
MSigDB: Molecular Signatures Database
MSC: Mesenchymal stem/stromal cells
N/C: Nuclear to cytoplasmic
NES: Normalized enrichment score
NF-κB: Nuclear factor kappa B
NHEJ: Nonhomologous End-Joining
PBS: Phosphate Buffered Saline
PCA: Principal Component Analysis
PGCC: Polyploid giant cancer cell
PI: Propidium iodide
PVDF: Polyvinylidene fluoride
RIN: RNA integrity number
RT: Room temperature
S-phase: Synthesis phase
SAC: Spindle Assembly Checkpoint
SDS‒PAGE: Sodium dodecyl sulfate‒polyacrylamide gel electrophoresis
TCGA: The Cancer Genome Atlas
TNF-α: Tumor necrosis factor alpha
TSGs: Tumor suppressor genes
WGD: Whole-genome doubling
